# PML modulates epigenetic composition of chromatin to regulate expression of pro-metastatic genes in triple-negative breast cancer

**DOI:** 10.1093/nar/gkad819

**Published:** 2023-10-12

**Authors:** Cristina Fracassi, Martina Ugge', Mohamed Abdelhalim, Ettore Zapparoli, Matilde Simoni, Daniela Magliulo, Davide Mazza, Dejan Lazarevic, Marco J Morelli, Philippe Collas, Rosa Bernardi

**Affiliations:** Division of Experimental Oncology, IRCCS San Raffaele Scientific Institute, Milano, Italy; Division of Experimental Oncology, IRCCS San Raffaele Scientific Institute, Milano, Italy; Department of Molecular Medicine, Institute of Basic Medical Sciences, Faculty of Medicine, University of Oslo, Oslo, Norway; Center for Omics Sciences, IRCCS San Raffaele Scientific Institute, Milano, Italy; Division of Experimental Oncology, IRCCS San Raffaele Scientific Institute, Milano, Italy; Division of Experimental Oncology, IRCCS San Raffaele Scientific Institute, Milano, Italy; Experimental Imaging Center, IRCCS San Raffaele Scientific Institute, Milano, Italy; Center for Omics Sciences, IRCCS San Raffaele Scientific Institute, Milano, Italy; Center for Omics Sciences, IRCCS San Raffaele Scientific Institute, Milano, Italy; Department of Molecular Medicine, Institute of Basic Medical Sciences, Faculty of Medicine, University of Oslo, Oslo, Norway; Department of Immunology and Transfusion Medicine, Oslo University Hospital, Oslo, Norway; Division of Experimental Oncology, IRCCS San Raffaele Scientific Institute, Milano, Italy

## Abstract

The promyelocytic leukemia (PML) protein organizes nuclear aggregates known as PML nuclear bodies (PML-NBs), where many transcription factors localize to be regulated. In addition, associations of PML and PML-NBs with chromatin are described in various cell types, further implicating PML in transcriptional regulation. However, a complete understanding of the functional consequences of PML association to DNA in cellular contexts where it promotes relevant phenotypes is still lacking. We examined PML chromatin association in triple-negative breast cancer (TNBC) cell lines, where it exerts important oncogenic functions. We find that PML associates discontinuously with large heterochromatic PML-associated domains (PADs) that contain discrete gene-rich euchromatic sub-domains locally depleted of PML. PML promotes heterochromatic organization in PADs and expression of pro-metastatic genes embedded in these sub-domains. Importantly, this occurs outside PML-NBs, suggesting that nucleoplasmic PML exerts a relevant gene regulatory function. We also find that PML plays indirect regulatory roles in TNBC cells by promoting the expression of pro-metastatic genes outside PADs. Our findings suggest that PML is an important transcriptional regulator of pro-oncogenic metagenes in TNBC cells, via transcriptional regulation and epigenetic organization of heterochromatin domains that embed regions of local transcriptional activity.

## Graphical Abstract

**Graphical Abstract:**
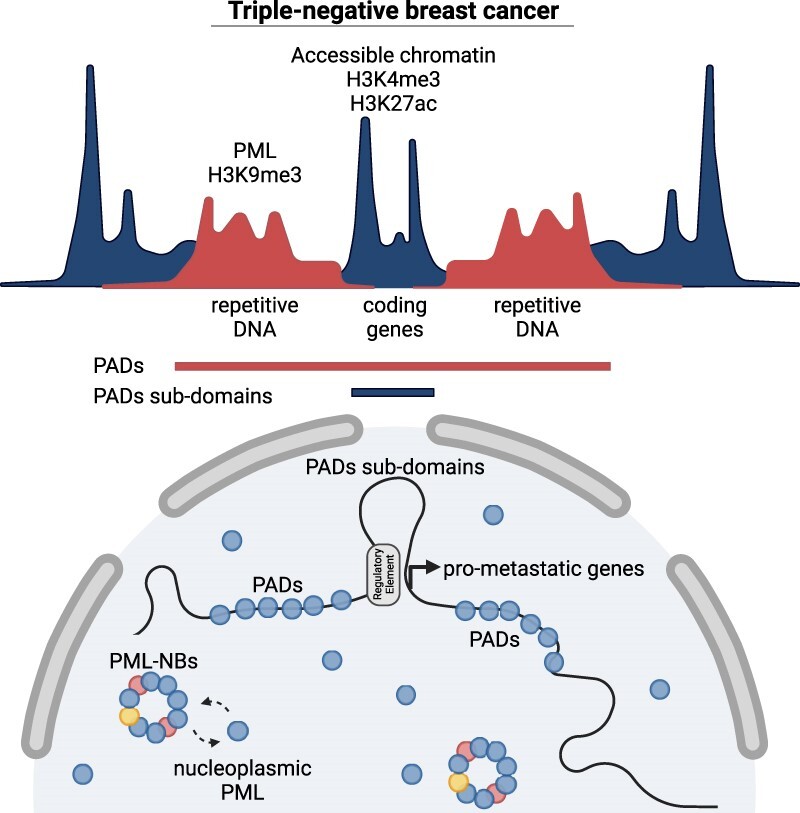

## Introduction

The PML (promyelocytic leukemia) nuclear protein provides a molecular scaffold for the nucleation of insoluble biomolecular condensates named PML nuclear bodies (PML-NBs). PML-NBs are generally described as platforms for the regulation of proteins therein associated upon cellular stress (1,2). However, imaging and biochemical fractionation studies show that PML actively shuttles between the nucleoplasm and PML-NBs (3–6), and it remains to be established whether PML exerts specific functions in these distinct compartments.

The molecular functions of PML are challenging to delineate due to a lack of enzymatic activities and well-defined functional domains and have been mostly assigned based on protein interactors. The predominance of transcription factors (TFs), transcriptional regulators and chromatin remodeling proteins in the PML interactome has long implicated PML in the regulation of transcription. Until recently, PML-mediated transcriptional regulation had been mostly linked to events occurring within PML-NBs, such as TFs post-translational modifications, and sequestration or assembly of transcriptional complexes (7). However, more recent evidence indicates that PML also associates with specific DNA regions to regulate transcription (8). Attempts to characterize PML-associated chromatin at a genome scale have mostly focused on identifying DNA associated with PML-NBs (9–11). These studies have mapped tissue-specific distribution of PML on chromatin with a limited overlap with PML-regulated transcriptomes (9–11). In addition, in mouse embryonic fibroblasts (MEFs) PML associates with large heterochromatic domains named PML-associated domains or PADs, where PML promotes the deposition of H3K9me3. Strikingly, PADs have been shown to be distant from PML-NBs (12). However, it remains unknown whether such PML-chromatin association impacts transcriptional regulation and bears any cell type-specific significance.

The transcriptional functions of PML are of particular interest in cancer, where PML has been described as a tumor suppressor or oncogene depending on tumor contexts (13). We and others have demonstrated that PML is overexpressed in triple-negative breast cancer (TNBC) where it displays oncogenic functions by promoting pro-tumoral metabolism, cancer stem cell maintenance and metastasis (14–17). However, the molecular determinants of PML-mediated transcriptional regulation in TNBC remain largely unknown.

Here, we mapped PML chromatin association in TNBC cells to evaluate its impact on transcriptional regulation. We find that PML associates with large heterochromatic regions outside PML-NBs, where it regulates deposition of H3K9me3. Importantly, these regions contain spatially restricted euchromatic domains where PML regulates expression of pro-metastatic genes. Our data suggest that in TNBC cells, PML acts as regulator of a metastasis gene expression program by modulating heterochromatin homeostasis.

## Materials and methods

### Cell culture, lentiviral vectors, lentiviral production and transduction

MDA-MB-231, BT549, MDA-MB-468 and MCF7 cells were purchased from ATCC and maintained in DMEM supplemented with 10% fetal bovine serum (FBS) (Euroclone) and 1% Penicillin/Streptomycin antibiotics (Lonza) at 37°C in a humidified atmosphere containing 5% CO_2_. For *in vitro* treatment with arsenic trioxide (ATO), cells were incubated for 6 and 24 h with 1 μM arsenic trioxide (Sigma-Aldrich A1010).

Third generation lentivirus (LV) stocks were prepared, concentrated and titrated as previously described (18,19). Briefly, self-inactivating (SIN) LV vectors were produced by transient transfection of HEK293T cells with the packaging plasmid pMDLg/pRRE, Rev-expressing pCMV-Rev, the VSV-G envelop-encoding pMD2.VSV-G plasmids, and shRNA-carrying vectors. Specifically, pLKO.1-puro vectors expressing shPML (TRCN0000003869, named shPML_01 and TRCN0000003868, named shPML_02) and shRNA control (SHC002, named shCTRL) were purchased from SIGMA (MISSION®). shPML_01 targets GTGTACGCCTTCTCCATCAAA, corresponding to position 1501 of the PML coding sequence; shPML_02 targets GTGTACCGGCAGATTGTGGAT, corresponding to position 493 of the PML coding sequence. Optimal puromycin concentration was pre-determined by performing dose-response curves and used at a final concentration of 2.5 μg/ml for MDA-MB-231 and MDA-MB-468 and of 1 μg/ml for BT549.

### Immunoblotting and immunoprecipitation

Proteins were extracted with RIPA buffer (Tris–HCl pH 8 50 mM, NaCl 150 mM, NP-40 1%, Na-deoxycholate 0.5%, SDS 0.1%), supplemented with protease inhibitors (Complete EDTA-free Protease Inhibitor Cocktail Tablets; Roche) and sonicated. Protein concentration was determined via Bradford assay. 10 μg of proteins were diluted in 4× Laemmli buffer and β-mercapthoetanol and boiled 5 min at 95°. To visualize PML after ATO treatment, equal cell numbers were lysed in 4× Laemmli buffer and β-mercapthoetanol, sonicated and boiled. Nuclear fractionation of MDA-MB-231 cells was performed using the Nuclear Extract Kit (ab219177, Abcam). Immunoprecipitation was performed on nuclear extracts as follows: MDA-MB-231 cell pellets were washed with 5 volumes of hypotonic buffer (HEPES 10 mM, MgCl_2_ 1.5 mM, KCl 10 mM, DTT 0.5 mM) and centrifuged at 1250 rpm 5 min at room temperature (RT). Resulting pellets were resuspended in 3 volumes of hypotonic buffer and incubated 10 min on ice. Triton X-100 was added to a final concentration of 0.5%, followed by 10 x Dounce homogenization with a tight pestle and 4°C 14 000 × g centrifugation for 1 min. Nuclear pellets were resuspended in extraction buffer (Tris–HCl 50 mM pH 7.6, NaCl 300 mM, NP-40 0.5%, glycerol 20%) and incubated 30 min at 4°C on rotation. 2 mM MgCl_2_ and 75 U/ml benzonase (Sigma Aldrich e1014-25ku) were added for 40 min at 4°C on rotation. Nuclear extracts were obtained upon 16 000 × g centrifugations at 4°C for 30 min. 3000 μg of nuclear extract was immunoprecipitated with 4 μg of anti-PML (Santa Cruz Biotechnology 71910) or negative IgG control (Jackson ImmunoResearch, 015000003) overnight at 4°C on rotation. 80 μl Sepharose beads (Cytiva Protein G Sepharose 4 Fast Flow, 17061801) were used for 4 h at 4°C on rotation. Beads were washed 3× in Co-IP buffer (NaCl 150 mM, Tris–HCl pH 7.5 50 mM, NP-40 0.5%) and elution was performed at 95°C for 5 min in 4X Laemmli buffer and β-mercapthoetanol. Input represents 3% of immunoprecipitated lysate. All buffers were supplemented with protease inhibitors (Complete EDTA-free Protease Inhibitor Cocktail Tablets; Roche). SDS-PAGE was performed at different polyacrylamide concentrations (4–15%) and transferred on polyvinylidene difluoride membrane through transBlot Turbo Transfer System (Bio-Rad). Membranes were blocked with 5% Milk or 5% BSA in PBS 0.1% Tween 20 and incubated with antibodies against PML (1:1000; Novus Biologicals 100-59787), LMNB1 (1:5000; Abcam 16048), H3K9me3 (1:10000; Abcam 8898), H3K27me3 (1:1000, Sigma-Aldrich 07–449), H3K4me3 (1:1000, Cell Signaling Technologies C42D8), H3K27ac (1:1000, Cell Signaling Technologies D5E4), β-Actin (1:10000, Santa Cruz Biotechnology 69879) and histone H3 (1:20000, Abcam 1791), SETDB1 (1:1000, ThermoFisher Scientific MA5-14960). Proteins were detected with peroxidase-conjugated antibodies (Normal IgG-HRP, 1:10000, Santa Cruz Biotechnology 2005/2357) using the ECL Western Blotting Detection Reagents (GE Healthcare).

### Immunofluorescence (IF) and DNA fluorescence *in situ* hybridization (Immuno DNA-FISH)

Cells were seeded at 50% confluence on coverslips in 12 well plates. After 24 hr, immuno DNA-FISH was performed as described (20). Fluorescently labelled probes specific for two chromosomal regions in PADs (CHR12: 11338598–11339543, Locus: 12p13.2, RP11-319K20; CHR11: 55926869–55927793, Locus: 11q12.1, RP11-294G10) were purchased form Empire Genomics and hybridized using the provided buffer solution. Immunostaining was performed after DNA-FISH. Blocking was performed in PBS, 0.05% Tween 20, 1% FBS for 30 min at RT and cells were incubated with antibodies against PML (1:200; Santa Cruz Biotechnology 966), diluted in PBS, 0.05% Tween 20, 1% FBS, for 1 h at RT. Next, secondary antibody (1:500, ThermoFisher scientific Alexafluor 488) diluted in PBS, 0.05% Tween 20, 10% FBS was used for 1 hr at RT. DNA was counterstained with DAPI and mounting was done in Mowiol.

### Proximity ligation assay (PLA)

Cells were seeded at 50% confluence on coverslips in 12-well plates. After 24 h, cells were fixed with 4% PFA for 10 min at RT and permeabilized with PBS and 1% Triton X-100 for 5 min. PLA was performed using the Duolink® Proximity Ligation Assay (Sigma-Aldrich) following the manufacturer's instructions. When PLA was coupled with IF, PLA was performed until the last wash in the Duolink buffer B 1×, then IF was performed as previously described. In order to test antibodies specificity, we included technical and biological controls: (i) omission of each probe separately; (ii) titration of primary antibodies; (iii) use of PML knockdown cells. Final antibodies concentrations: anti-PML (1:1000; Santa Cruz Biotechnology 966), anti-H3K9me3 (1:10000; Abcam 8898) and anti-SETDB1 (1:200, ThermoFisher scientific MA5-14960).

### Microscopy, imaging and quantification

Images were acquired with the GE HealthCare DeltaVision Ultra microscope (60× objectives) in z-stacks (stacks of 0.2 μm). Identical settings and contrasts were applied for all images of the same experiment to allow data comparison. Raw images were analyzed with Fiji software (21). Colocalization analysis among fluorescent signals were performed by manual counting the number of colocalization occurring in each nucleus and on the same z-stacks. 3D distance (in μm) among PML and DNA-FISH foci fluorescent signals was measured by localizing the foci in the two channels using FISHquant (22) and then calculating the distance between nearest neighbors using a custom written Matlab script (provided in Supplementary file 2).

### Quantitative real time PCR (qRT-PCR)

Total RNA was isolated using RNeasy mini Kit (Qiagen). Equal amounts (1 μg) of RNA were reverse transcribed into cDNA with Advantage RT-for-PCR Kit (Clontech) and analyzed by qPCR using a 7900 Fast-Real Time PCR System (Applied Biosystems). Probes for TaqMan assays were purchased from Applied Biosystems and use as follows: PLOD2 HS01118190_m1; TNC HS011156665_m1; SPARC HS00234160_m1; GAPDH HS02758991_g1; LOX HS00942480_m1; ZEB2 HS00207691_m1; PML HS00971694_m1; CIITA HS00172106_m1. Data were normalized to the *GAPDH* gene. Relative expression was calculated using the comparative threshold cycle method (2-ΔΔCt), except for assessing basal gene expression where the 2-ΔCt was used.

### RNA-sequencing (RNA-seq)

Total RNA was isolated from replicate experiments using the RNAeasy mini kit (Qiagen) and processed for Illumina library preparation. TruSeq stranded mRNA protocol (Illumina) was used for 5′/3′ library preparation. Libraries were then barcoded, pooled and sequenced on an Illumina Nova-Seq 6000 sequencing system. For each run, RNA sequencing experiments were performed generating 30M single end reads, 100 nucleotide long. Sequencing adapters were removed using trimmomatic v0.39 (https://github.com/usadellab/Trimmomatic/releases/tag/v0.39) (23), and fastq files were then aligned to the human genome assembly GRCh38 (hg38) using the STAR aligner v2.5.3a (https://github.com/alexdobin/STAR/releases/tag/2.5.3a) (24). Annotation of genomic features was performed using the featureCounts tool v1.6.4 (25) using the GENCODE v31 Gene transfer format (GTF). Differential gene expression was evaluated in R/BioConductor using the DeSeq2 package (26) and using a false discovery rate (FDR) of 0.05 for significance.

### Chromatin-immunoprecipiation (ChIP)

Cells were seeded 24 h before chromatin isolation at 60–70% confluence in 15 cm plates. Cells were trypsinized and double crosslinking was performed in suspension. Cells were first resuspended in PBS containing 2 mM Di(N-succinimidyl) glutarate (DSG, Sigma-Aldrich 80424) for 45 min at RT on gentle rotation. Cells were then centrifuged at 1200 rpm at RT for 5 min. The resulting pellet was resuspended in PBS containing 1% Formaldehyde (Sigma-Aldrich 252549) for 10 min at RT on gentle rotation. After formaldehyde quenching with Glycine (final concentration 125 mM) for 5 min, cells were centrifuged at 1350 × g at 4°C for 5 min, and the supernatant was discarded. Chromatin extraction was performed as previously described (27) and sonication was performed using the Bioruptor (Diagenode Bioruptor 300) at high intensity, 30 s ON and 40 s OFF for 12 cycles, to obtain chromatin enriched in fragments of 200–1500 bp. Crosslinking was reversed, and samples were purified using QIAquick PCR purification kit (Qiagen). Chromatin was then quantified with Nanodrop spectrophotometer and efficiency of sonication was measured by agarose gel electrophoresis. For sequencing, DNA quality was evaluated with a High Sensitivity D5000 ScreenTape (Agilent Technologies). After sedimentation, 25 or 50 μg (for sequencing) of chromatin were incubated with 20 μg of anti-PML (Santa Cruz Biotechnology 71910), 12.5 μg of STAT3 (Cell Signaling Technologies 124H6) and 72 ng of anti-SETDB1 (ThermoFisher scientific MA5-14960). 10 μg of chromatin with 5 μg of anti-H3K9me3 (Abcam 8898), H3K27me3 (Abcam 6002), H3K4me3 (Cell Signaling Technologies C42D8) and H3K27ac (Cell Signaling Technologies D5E4) and 12.5 μg of anti-LMNB1 (Abcam 16048). Normal mouse and rabbit IgG were used as negative control (Santa Cruz Biotechnology sc2025 and sc2027). For each ChIP, 5% of chromatin was collected as input sample. Chromatin was pre-coupled to target-antibody overnight at 4°C on rotation and the day after beads were added (ChIP-IT Protein G magnetic beads 53033 or Magna ChIP™ Protein A Magnetic Beads 16–661) for 4 h at 4°C on rotation. ChIP samples were washed three times in ice-cold RIPA buffer + 1% SDS (Tris–HCl pH 8 10 mM, NaCl 140 mM, Triton X-100 1%, Na-deoxycholate 0.1%, EDTA 1 mM, EGTA 0.5 mM) and elution was performed in 250 μl of Elution buffer (NaCl 50 mM, Tris–HCl pH 7.5 20 mM, EDTA 5 mM, SDS 1%, RNAse-A 0.5 μg/ml, Proteinase K 2 μg/ml) for 6 hr at 37°C on rotation. Finally, beads were removed, and samples were de-crosslinked overnight at 65°C. DNA was purified with the QIAquick PCR purification kit (Qiagen) and used for qPCR analysis or library preparation. qPCR was performed by using the SsoAdvanced Universal Probes Supermix (Bio-Rad) and a 7900 Fast-Real Time PCR System (Applied Biosystem). Enrichment levels were expressed as signal over background and normalized enrichment levels upon PML silencing were expressed as fold over control.

All primers have been manually designed and purchased by Eurofins. PAD_01 forward, ACTAAGCCACGAGCTGC; PAD_01 reverse, TATCTTCGAGCCTGGGA; PAD_02 forward, CAGGACTTGTAGGAGACCAG; PAD_02 reverse, GCACACAGGCCTGGTTAT; PAD_03 forward, GAGGTCATAAGGGAATGTTG; PAD_03 reverse, CGATTATGGTGGTATGGC; PAD_04 forward, TTCAGCACACAAGTTCAGG, PAD_04 reverse, AATCTGACCTCAGCCTGTC; HHAT forward, TGTGCTCCTGCACTGTG; HHAT reverse, AGTCACCAAGGACAGCTTC; CIITA forward, CACTGACATAGAAAGGGGTCC; CIITA reverse, TTATGCTGTTATGCAACTTGACTCT; SPARC forward, AGGCAAAGAGAGACTGTGAAAGA; SPARC reverse, CCAGTGTACCTGTCCTTGCT; LOX forward, AGGTCACACTGGAAATTTGTCT; LOX reverse, CAATGCCTGCTCTGTGTCCT.

### ChIP-sequencing (ChIP-seq) and ATAC-sequencing (ATAC-seq)

ATAC-seq was performed as reported (28) and libraries were sequenced on Illumina platforms generating 40M reads, 50 nucleotide long, in paired end. For ChIP-seq libraries were constructed following the ChIPSeq Illumina protocol (Illumina). Libraries were barcoded, pooled and sequenced on an Illumina Nova-Seq sequencing system. ChIP-seq experiments were performed generating 40M reads, 100 nucleotide long, in paired end. After sequencing, reads were trimmed using BBDuk from BBTools suite version 37.36 (http://sourceforge.net/projects/bbmap/) with suggested settings (ktrim = r k = 23 mink = 11 hdist = 1), then mapped using BWA-MEM version 0.7.12-r1039 (29) on the human genome assembly GRCh38 (containing only autosomes). Uniquely mapped reads were selected with MarkDuplicates from Picard Tools version 1.104 (http://broadinstitute.github.io/picard/). Further filtering was done on reads mapping in regions present in the ENCODE hg38 blacklist (30) and regions flagged as not primary alignment or with mapping quality score less than 15.

For ATAC-seq, H3K4me3 and H3K27ac ChIP-seq peaks were detected using MACS2 v2.2.7.1 (https://github.com/macs3-project/MACS/releases/tag/v2.2.7.1) (31). ChIP and ATAC read counts were normalized to library size using the *reads per genome coverage* (RPGC) function in Deeptools v3.5.1 (https://github.com/deeptools/deepTools/releases/tag/3.5.1) (32). Bigwig tracks of PML, H3K9me3, H3K27me3 and LMNB1 ChIP-seq were generated from Log2(Chip/Input) ratios in 1-kb bins using bamCompare from Deeptools (32). Bigwig files for normalized read counts or Log2 (Chip/Input) ratios were visualized using Integrative Genomics Viewer (https://software.broadinstitute.org/software/igv/) (33). To assess ChIP data quality and reproducibility, Pearson correlations were determined between replicates from Log2(ChIP/Input) ratios or normalized counts in 1-kb bins across the genome using Deeptools (32).

Diffbind (version 3.10.0, 33) was used to identify differentially accessible regions (peaks) from ATAC-seq peaks. Peaksets from MACS2 and associated metadata for each sample were read into Diffbind within R and EdgeR (34) was employed within Diffbind for the identification of peaks which were differentially accessible between the PML silenced and control MDA-MB-231 cells (*P* < 0.05, FDR < 0.05, FC>/<0.5).

### Mapping PADs, LADs and H3K9me3 domains

For PML, LMNB1 and H3K9me3 ChIP-seq normalization bias were avoided by down-samplings for each chromosome each pair of mapped ChIP and input read files. Mapped reads were used to call domain using ten runs of Enriched Domain Detector (EDD; http://github.com/CollasLab/edd) (35) with auto-estimation of GapPenalty and BinSize, and mean GapPenalty and BinSize values from these runs were used for a last run. Final domains were the union of domains of all replicates.

### Intersections and overlaps

Intersects were determined using BEDTools v2.30 (https://github.com/arq5x/bedtools2/releases/tag/v2.30.0) (36) and BEDOPS v2.4.41 (https://github.com/bedops/bedops/releases/tag/v2.4.41) (37). The number of overlapping peaks/domains between different conditions was computed with the Intervene v0.5.8 *venn* function (https://github.com/asntech/intervene/releases/tag/intervene-v0.5.8) (38). Randomized domains or peaks were computed with the *bedtools shuffle* function, maintaining the same size and number of the input genomic sequences. Inter PADs regions and PADs subdomains where obtained with the *bedops –not-element-of* function. Gene annotation of ATAC-seq, H3K4me3 and H3K27ac peaks was performed with GREAT (39), while, genes were ascribed to an EDD called domain if they overlapped with a domain by at least one base-pair. Expected partition distribution plots for ATAC differential peaks was performed with the Genomic distribution package (40). Mean Log2(ChIP/Input) or normalized counts from all replicates were calculated using wiggletools v1.2 (https://github.com/Ensembl/WiggleTools). Mean Log2(ChIP/Input) or normalized counts of the PADs intersected and non-intersected regions were calculated using *multiBigwigSummary* from Deeptools.

### Relative distance measurements

We calculated a relative distance metric with BEDtools (*reldist* function) (36) using ATAC differential peaks as the ‘a’ arguments and differentially expressed genes or randomized ATAC differential peaks as the ‘b’ arguments. We compared the distribution of relative distances using a Kolmogorov–Smirnov test.

### Functional enrichment analysis

Gene set enrichment analysis was performed with the EnrichR webtool (41). Reactome, Hallmark for cancer, Biocarta and GO biological process were used as reference databases and significant pathways were filtered by adjusted *P*-value <0.1. Enrichment of genomic features annotated in PADs or enhancer sequences was assessed via fisher's exact test using a collection of UCSC feature tables and transcription factors binding sites form ENCODE provided by the LOLAweb database (42). ggplot2 in R (https://ggplot2.tidyverse.org/) (43) was used for plots.

### Statistical analysis

Data were processed using GraphPad Prism version 9.0.2 (GraphPad Software, San Diego, California, USA, www.graphpad.com), and the R statistical environment. Permutation tests were performed on R using the Bioconductor packages RegioneR (version 3.14).

## Results

### PML associates with large heterochromatic regions in TNBC cells

**Figure 1.**
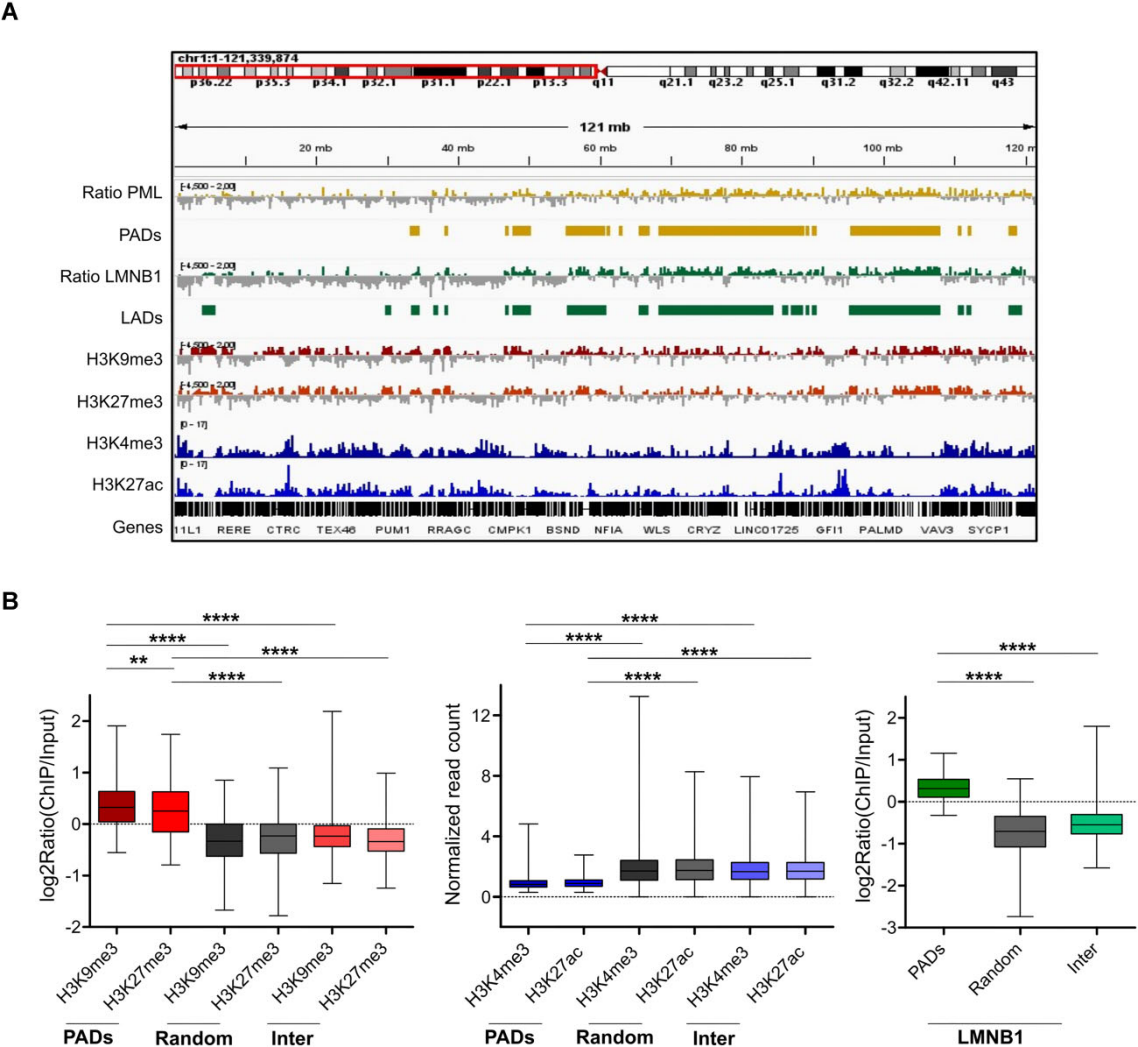
PML associates with heterochromatic domains in TNBC. (**A**) Genome browser view of Log2(ChIP/input) ratios and normalized counts (*y* axis range shown in brackets) of PML, LMNB1, H3K9me3, H3K27me3, H3K4me3, H3K27ac, and called PADs (PML-associated domains) and LADs (lamin-associated domains). (**B**) Enrichment in PADs, random-PADs (Random) and inter-PADs regions (Inter) of H3K9me3 and H3K27me3 (left graph), H3K4me3 and H3K27ac (middle graph), LMNB1 (right graph); bar, median; whiskers, min-max; **P < 10^−2^, ****P < 10^−4^; unpaired t-tests with Welch's correction. Data were obtained in MDA-MB-231 cells.

We have previously demonstrated that in TNBC cells PML promotes a metastatic phenotype that is mediated, at least in part, by transcriptional regulation of HIF1α-target genes (16). Given the multifaceted function of PML in this tumor context (14–17), we aimed to provide mechanistic insights into PML transcriptional regulation at a whole genome level. First, we mapped PML chromatin association in the TNBC cell line MDA-MB-231. Because PML is not a DNA-binding protein, to stabilize its association with chromatin we utilized a two-step protein-protein and protein-DNA crosslinking protocol initially described for ChIP-seq of transcriptional co-regulators (44). PADs were identified with Enriched Domain Detector (EDD; Figure 1A) (35) from the union of 3 biological replicates (Pearson correlations of PML enrichment profiles (log2(ChIP/Input)) shown in Supplementary Figure S1A, B). We identified 421 PADs of median length 2.3 mb distributed throughout the genome and encompassing a total of 970 mb (Supplementary Table S1). Given the low chromatin occupancy of PML over input (Figure 1A, Supplementary Figure S1A, B), we measured enrichment of PML with respect to control IgG in 4 representative PADs and confirmed significant DNA association (Supplementary Figure S1C). Moreover, PML silencing with two independent shRNAs (Supplementary Figure S1D, E) confirmed specific PML association to 4 PADs, and not to an inter-PAD region (*HHAT*, Supplementary Figure S1F). Although off-target effects of RNAi approaches have been described (45), rescue experiments with siRNA-resistant PML cDNA were not performed because of multiplicity of PML splicing isoforms and possible artefactual results upon PML overexpression (46). As shRNAs used in this study target N-terminal regions that are common to PML splicing isoforms, it is presently unknown whether a specific protein isoform may be enriched at PADs.

Importantly, PML association to representative PADs was validated in two additional TNBC cell lines (BT549 and MDA-MB-468 cells, representative of mesenchymal and basal-like 1 subtypes respectively, while MDA-MB-231 cells represent mesenchymal stem-like subtype) (47), while no enrichment over IgG was observed in ER + breast cancer cells MCF7 (Supplementary Figure S2A–D), indicating that PADs are not universal.

To characterize the epigenetic profile of PADs, we mapped heterochromatic (H3K9me3 and H3K27me3) and euchromatic (H3K4me3 and H3K27ac) histone modifications by ChIP-seq (Supplementary Figure S3A–D). We found that PADs are enriched in H3K9me3 and H3K27me3 compared to randomized domains of same size and number and all inter-PAD regions (Figure 1A, B), but not in H3K4me3 and H3K27ac (Figure 1A, B). In addition, PADs are gene-poor as compared to inter-PAD regions and contain mostly non-protein-coding genes (Supplementary Table S1).

Many large H3K9me3-rich heterochromatic regions are defined by association with nuclear lamins (lamina-associated domains or LADs) (48). In mapping lamin B1 (LMNB1) chromatin association by ChIP-seq (Supplementary Figure S3A, B), we found that PADs and LADs share similar features (Supplementary Table S1), and that LMNB1 is enriched in PADs (Figure 1A, B). Accordingly, most PADs overlap with LADs (Supplementary Table S2). This was confirmed with the LOLAweb database (42), which identified LMNB1-associated DNA within the highest ranked enriched features in PADs (Supplementary Figure S3E). Moreover, PADs are enriched in repetitive elements (Supplementary Figure S3E, F). Notably, repeats typical of heterochromatin, such as long interspersed nuclear elements (LINEs) and long terminal repeats (LTRs), are enriched in PADs relative to random DNA regions, while repeats typically found in active chromatin, such as short interspersed nuclear elements (SINEs), are not (Supplementary Figure S3G).

Interestingly, although most PADs overlap with LADs, unique domains of LMNB1 or PML association with distinct epigenetic composition were identified (Supplementary Figure S4A). Specifically, unique LADs (U-LADs) are more heterochromatic than unique PADs (U-PADs) and domains of PML/LMNB1 association (Common), while U-PADs are more euchromatic and gene-rich than both U-LADs and Common domains (Supplementary Figure S4B–D). These data indicate that PML confers qualitative differences to regions of LMNB1 association.

**Figure 2.**
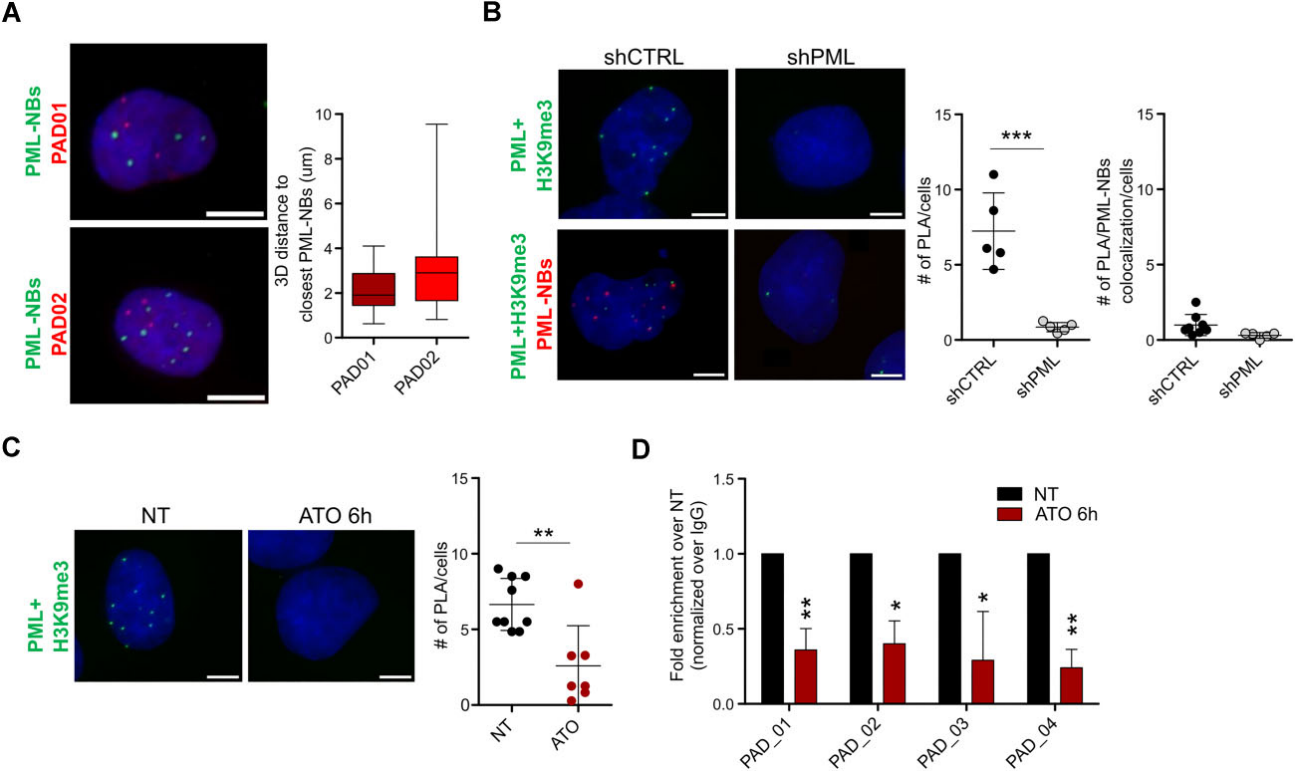
PML association with heterochromatin does not occur at PML-NBs. (**A**) Left panels, immuno-DNA-FISH of PML (green signal, PML-NBs) and PADs (DNA-FISH probes, red signal). Graph on the right indicates the distribution of 3D distances between FISH probes and the nearest PML-NB in μm. A total of 20 nuclei/conditions were analyzed. Data represent mean values ± SD. (**B**) Interaction of PML and H3K9me3 via proximity ligation analysis (green signal, upper panels) and proximity ligation analysis coupled to PML immunofluorescence (red signal, lower panels) in control (shCTRL) and PML silenced (shPML) cells. Graphs on the right indicate the number of PML/H3K9me3 interaction foci/cell and the number of PML/H3K9me3 interaction foci colocalizing with PML-NBs/cell. A total of 34 nuclei/condition were analyzed. Data represent mean values ± SD. Statistical significance was determined by unpaired Student's t-test. ***P < 10^−3^. (**C**) Interaction of PML and H3K9me3 via proximity ligation analysis (green signal) in untreated cells (NT) and cells treated with 1 μM arsenic trioxide (ATO) for 6 hours. Left panel, number of PML/H3K9me3 interaction foci/cell in NT and ATO-treated cells. A total of 40 nuclei/condition were analyzed. Statistical significance was determined by unpaired Student's t-test. **P < 10^−2^. (**D**) Association of PML to 4 intra-PAD sequences via ChIP-qPCR in NT and ATO-treated cells. Shown are mean values ± SD of 3 biological replicates normalized over IgG and represented as fold enrichment over NT. Statistical significance was determined by paired Student's t-test. *P < 5*10^−2^, **P < 10^−2^. All data were obtained in MDA-MB-231 cells and DNA was stained with DAPI (blue). Images are compressed z-stacks. Bar: 5 μm.

Finally, as previously observed in MEFs (12), immuno-FISH analysis revealed that PADs are not adjacent to PML-NBs (Figure 2A). This was confirmed by proximity ligation of PML and H3K9me3, a heterochromatic mark of PADs (Figure 1B). The majority of PML/H3K9me3 specific interaction foci (on average 7 foci/cell) did not colocalize with PML-NBs (Figure 2B), and minimal colocalization of H3K9me3 foci and PML-NBs was observed by immunofluorescence (Supplementary Figure S5A). Our data are in line with previous reports suggesting that PML immunoprecipitation tends to enrich for soluble PML moieties rather than insoluble PML-NBs (8,9,49). To provide further evidence to our observations, we used arsenic trioxide, which promotes PML SUMOylation and concentration into PML-NBs before inducing PML degradation (Supplementary Figure S5B, C) (50,51). Short-term incubation with arsenic trioxide reduced PML/H3K9me3 interaction and hampered PML association to PADs (Figure 2C, D).

We conclude that PML associates with large heterochromatic domains enriched in LMNB1 and DNA repeats in TNBC cells. Further, although we cannot exclude that specific PADs are adjacent to PML aggregates, this association occurs largely outside PML-NBs.

### PML modulates heterochromatin distribution

**Figure 3.**
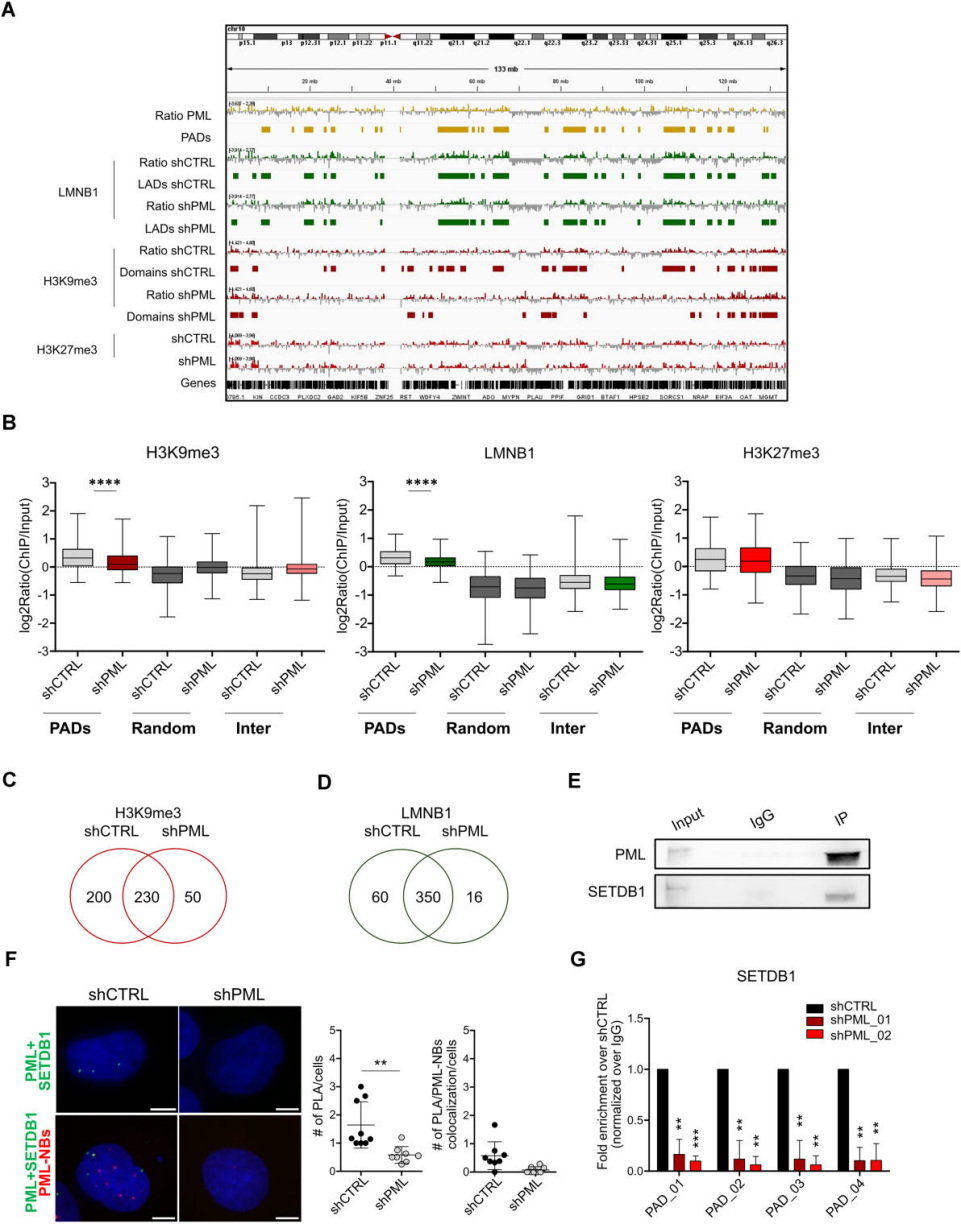
PML regulates the organization of H3K9me3 domains. (**A**) Genome browser view of Log2(ChIP/input) ratios (*y* axis range shown in brackets) of PML, LMNB1, H3K9me3, H3K27me3 and called PADs, LADs and H3K9me3 domains in control (shCTRL) and PML silenced (shPML) cells. (**B**) Enrichment in PADs, random-PADs (Random) and inter-PADs regions (Inter) of H3K9me3 (left graph), LMNB1 (middle graph) and H3K27me3 (right graph) in shCTRL and shPML cells; bar, median; whiskers, min-max; ****P < 10^−4^; unpaired t-tests with Welch's correction. (C, D) Venn diagrams of overlapping H3K9me3 (**C**) and LMNB1 (**D**) domains in control (shCTRL) and cells silenced for PML (shPML). (**E**) Immunoprecipitation with a PML-specific antibody or isotype control (IgG) and immunoblot with PML and SETDB1 antibodies in MDA-MB-231 cells. Input represents 3%. (**F**) Interaction of PML and SETDB1 via proximity ligation analysis (green signal, upper panels) and proximity ligation analysis coupled to PML immunofluorescence (red signal, lower panels) in control (shCTRL) and PML silenced (shPML) cells. Graphs on the right indicate the number of PML/SETDB1 interaction foci/cell and the number of PML/SETDB1 interaction foci colocalizing with PML-NBs/cell. A total of 40 nuclei/conditions were analyzed. DNA was stained with DAPI (blue). Images are compressed z-stacks. Bar: 5 μm. Data represent mean values ± SD. Statistical significance was determined by unpaired Student's *t*-test. **P < 10^−2^. (**G**) Association of SETDB1 to four intra-PAD sequences via ChIP-qPCR in cells silenced with a control shRNA (shCTRL) and two shRNAs against PML (shPML_01 and shPML_02). Shown are mean values ± SD of three biological replicates normalized over IgG and represented as fold enrichment over shCTRL. Statistical significance was determined by paired Student's *t*-test. **P < 10^−2^, ***P < 10^−3^. All data were obtained in MDA-MB-231 cells.

To elucidate the role of PML in heterochromatin maintenance and genome organization in PADs, ChIP-seq of H3K9me3, H3K27me3 and LMNB1 was performed in TNBC cells upon PML silencing (Supplementary Figure S3A, B). Reducing PML levels impaired enrichment of H3K9me3 but not H3K27me3 within PADs (Figure 3A, B). Of note, total levels of H3K9me3 were unchanged upon PML silencing (Supplementary Figure S6A), suggesting that decreased H3K9me3 enrichment at PADs may be accompanied by ectopic deposition. Thus, genome distribution of H3K9me3 was further explored by calling H3K9me3 domains in PML-silenced and control TNBC cells (Figure 3A). We identified 430 H3K9me3 domains in control cells, of which 70% overlapped with PADs (Supplementary Table S2; Supplementary Figure S6B). PML silencing led to a drastic decrease of H3K9me3 domains (Figure 3C, Supplementary Table S1), which occurred mostly within PADs (40% to 14% H3K9me3 coverage; Supplementary Table S2). Conversely, H3K9me3 domains outside PADs increased in cells with reduced PML expression (Supplementary Figure S6B), in line with our view of ectopic H3K9me3 deposition. These observations confirm that PML enables H3K9me3 deposition specifically within PADs.

PML silencing also led to reduced LMNB1 enrichment in PADs (Figure 3A, B; Supplementary Figure S6C) and reduced LADs numbers (Supplementary Table S1; Figure 3D), albeit less remarkably than H3K9me3 reduction. Interestingly, total LMNB1 protein levels were upregulated in PML-low cells (Supplementary Figure S6D), indicating that reduced LMNB1 genomic enrichment was not due to lower protein expression. Nuclear fractionation studies showed that LMNB1 accumulated in the nuclear soluble fraction in conditions of PML silencing (Supplementary Figure S6E), which is in line with LMNB1 enrichment at LADs being promoted by H3K9 methylation (52,53).

Finally, in search of a mechanistic function of PML in mediating heterochromatin deposition, we investigated the role of the H3K9 methyltransferase SETDB1, which plays oncogenic and pro-metastatic functions in TNBC (54) and interacts with PML in mouse embryonic cells (55). We confirmed that PML interacts with SETDB1 in TNBC cells (Figure 3E, F), albeit with lower frequency than PML/H3K9me3 interaction (Figure 2B). In agreement with previous observations with H3K9me3 and PADs DNA (Figure 2), few PML/SETDB1 interaction foci colocalized with PML-NBs (Figure 3F). Importantly, PML silencing impaired SETDB1 binding to four representative PADs (Figure 3G, Supplementary Figure S6F), suggesting that PML regulates H3K9me3 deposition in PADs by promoting DNA association of the histone methyltransferase SETDB1.

### Reduced PML levels provoke global increase and spreading of active chromatin marks

**Figure 4.**
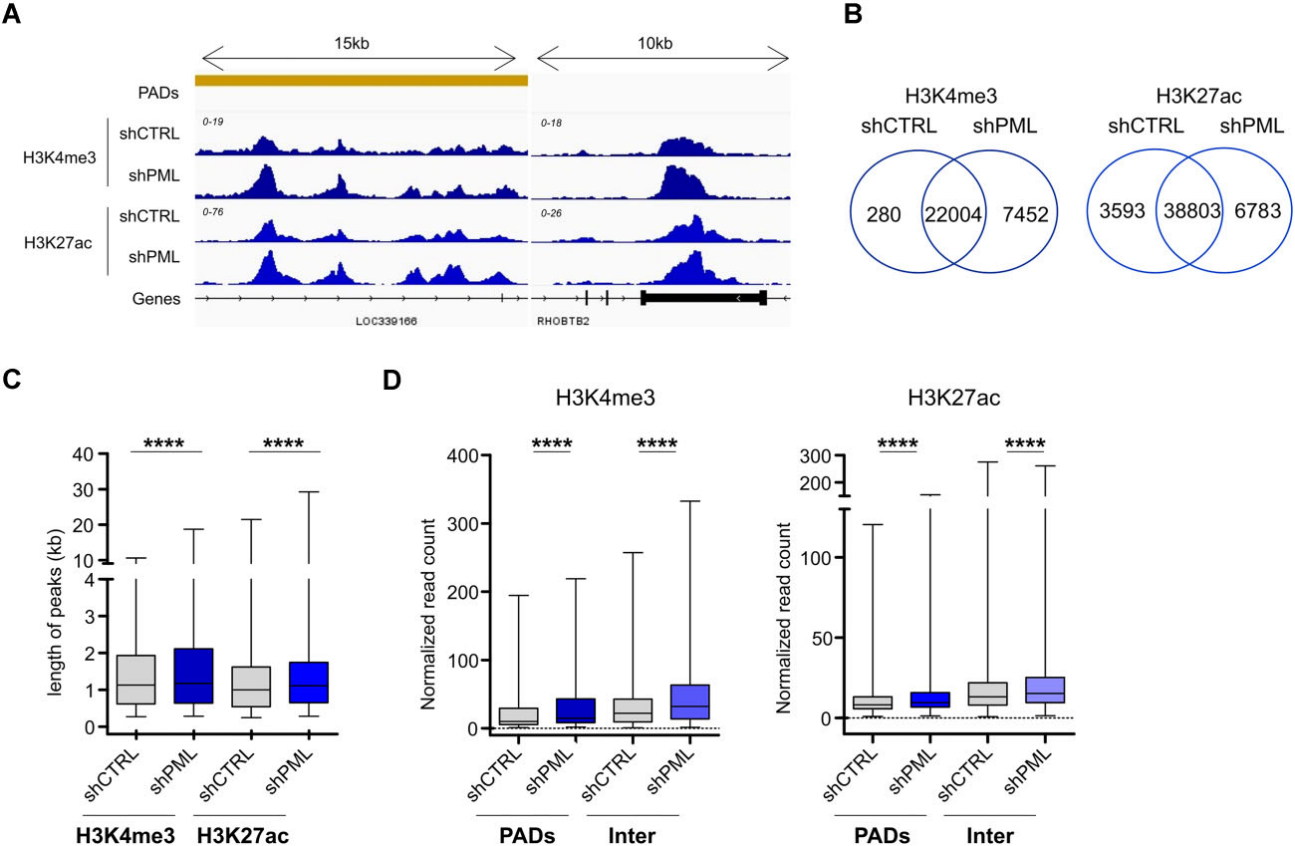
PML regulates spreading of H3K4me3 and H3K27ac in PADs and inter-PAD regions. (**A**) Genome browser view of H3K4me3 and H3K27ac enrichment (*y* axis range shown in brackets) on two randomly selected genes inside (left) and outside (right) PADs in control (shCTRL) and PML silenced (shPML) MDA-MB-231 cells. (**B**) Venn diagrams of overlapping H3K4me3 (left) and H3K27ac (right) peaks in control (shCTRL) and cells silenced for PML (shPML). (**C**) Lengths of H3K4me3 and H3K27ac peaks in shCTRL and shPML cells. (**D**) Enrichment of H3K4me3 and H3K27ac in PADs and inter-PADs regions (Inter) in shCTRL and shPML cells. For (C, D) bar, median; whiskers, min-max; ****P < 10^−4^; unpaired *t*-tests with Welch's correction. All data were obtained in MDA-MB-231 cells.

Heterochromatin and euchromatin domains subsist in a state of reciprocal equilibrium in the genome, such that disruption of one compartment may lead to spreading and reorganization of the other (56). With this in mind, we mapped two active chromatin marks, H3K4me3 and H3K27ac in TNBC cells upon PML silencing (Supplementary Figure S3C, D). Reduced PML expression led to genome-wide increase in the number and intensity of H3K4me3 and H3K27ac peaks (Figure 4A, B; Supplementary Table S1), which was not due to global changes in total H3 levels or post-translational modifications (Supplementary Figure S6G). Moreover, euchromatic peaks in PML-low cells showed increased length compared to control cells (Figure 4C), suggesting that silencing of PML promotes spreading of active chromatin marks. This increased enrichment occurred both within and outside PADs (Figure 4D), with a larger overall increase in the number of euchromatic peaks in inter-PAD regions than within PADs (Supplementary Figure S6H, I). We conclude that knocking-down PML in TNBC cells prompts deposition of euchromatic marks that is not restricted to PADs.

### PADs contain euchromatic regions that escape the repressive chromatin environment

**Figure 5.**
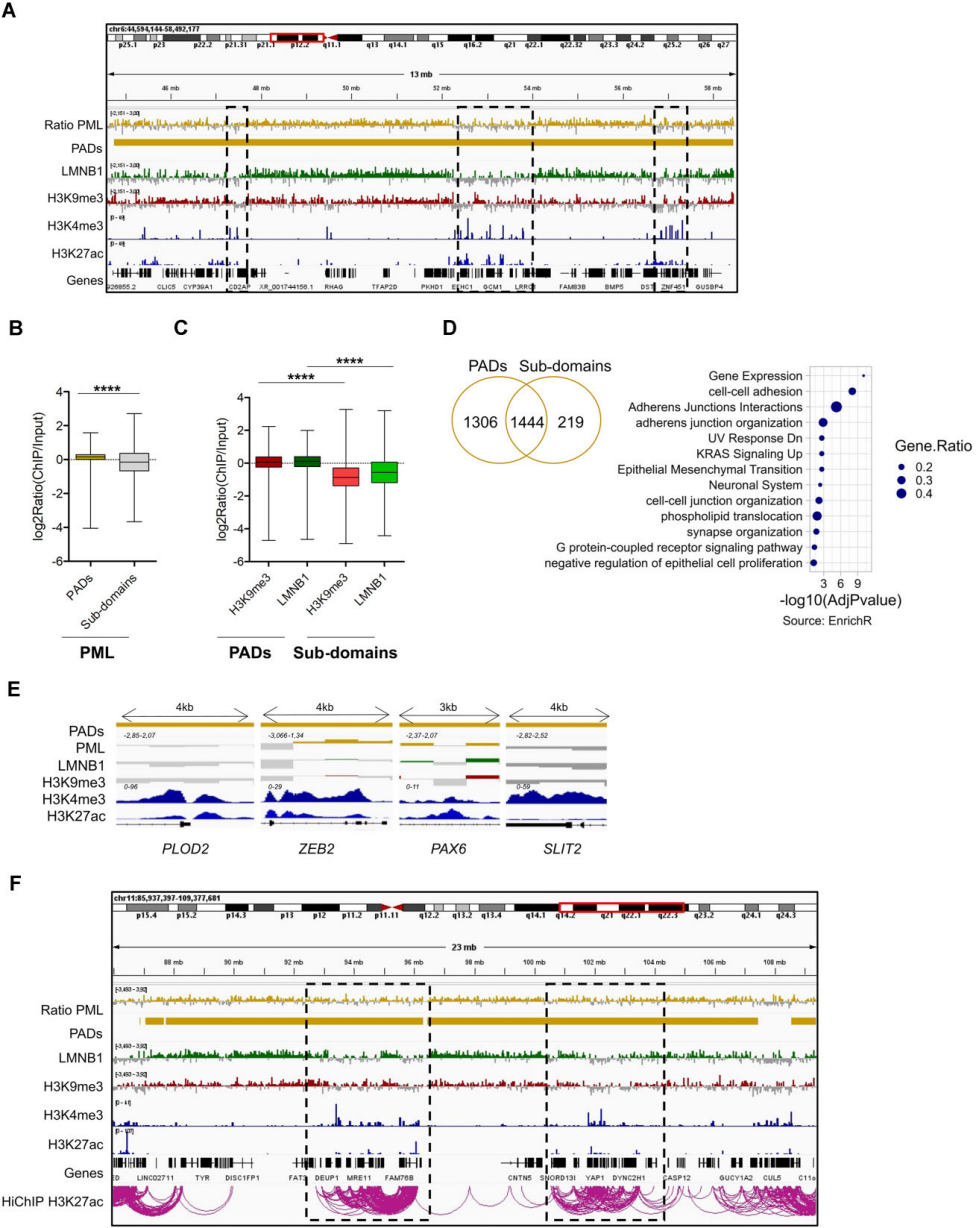
Euchromatic sub-domains escape the repressive environment of PADs. (**A**) Genome browser view of Log2(ChIP/input) ratios and normalized counts (*y* axis range shown in brackets) of PML, LMNB1, H3K9me3, H3K4me3, H3K27ac and called PADs. (B, C) Enrichment of PML (**B**), H3K9me3 and LMNB1 (**C**) in PADs and euchromatic sub-domains in PADs (sub-domains); bar, median; whiskers, min–max; unpaired *t*-tests with Welch's correction; ****P < 10^−4^. (**D**) Overlap of coding genes mapping with coding genes in PADs sub-domains (left Venn diagram). Gene set enrichment analysis (EnrichR webtool) of genes mapping to PADs subdomains. The Gene Ratio value is obtained dividing the number of observed genes for total genes contained in the indicated functional families. Results from the Fisher's exact test are represented as –log_10_(adjusted *P*-value). (**E**) Genome browser views of PADs and enrichment of PML, LMNB1, H3K9me3, H3K4me3 and H3K27ac at the promoter-proximal regions of the *PLOD2*, *ZEB2*, *PAX6*, *SLIT2* genes. (**F**) Genome browser view of Log_2_(ChIP/input) ratios and normalized counts (*y* axis range shown in brackets) of PML, LMNB1, H3K9me3, H3K4me3, H3K27ac, called PADs and H3K27ac HiChIP loops. All data were obtained in MDA-MB-231 cells.

The increase of euchromatin marks within PADs after PML silencing (Figure 4) prompted us to define in more detail the features of active chromatin regions in PADs. Small euchromatic regions within PADs were identified by H3K4me3 peaks in control TNBC cells (Figure 5A). Interestingly, PML binding frequency was lower in these regions than in remaining PADs (Figure 5A, B), arguing that PADs are domains of non-uniform PML interaction frequencies.

A similar pattern of LMNB1 coverage was described in LADs, where local euchromatic and lamin-depleted sub-domains were found to contain differentially expressed genes (53,57). Similarly, local euchromatic and PML-depleted sub-domains (named PADs sub-domains) were depleted of H3K9me3 and LMNB1 compared to the rest of PADs (Figure 5A, C), suggesting evasion from the overall heterochromatic state. Notably, 52% of the coding genes mapping to PADs are contained within PADs sub-domains (Figure 5D, with a density of 512 genes/mb as opposed to 11 genes/mb of PADs overall: Supplementary Tables S1, S3 and S4). Gene set enrichment analysis revealed that these genes cluster in families with annotations linked to cell migration and metastasis (*e.g*. cell adhesion and epithelial to mesenchymal transition; EMT) along with regulation of the neuronal system (Figure 5D). Included in these families are known PML-regulated genes involved in neural development and axon guidance, such as *PAX6* and *SLIT2* (Figure 5E) (58,59). Importantly, metastasis-promoting genes that we previously described as regulated by PML in TNBC cells (16) were also contained in these regions. These include *PLOD2*, an enzyme involved in extracellular matrix organization, and *ZEB2*, a master regulator of EMT (Figure 5E). As expected, promoters of these genes were enriched in H3K4me3 and H3K27ac and depleted of H3K9me3, LMNB1 and PML (Figure 5E), confirming that they evade the heterochromatic configuration of PADs. In addition, by utilizing a deposited MDA-MB-231 HiChIP dataset targeting H3K27ac to identify enhancer-promoter linkages (60), we observed that PADs sub-domains are sites of productive interactions between regulatory regions (Figure 5F), suggesting a state of localized active transcription.

### PML regulates expression of pro-metastatic genes in TNBC via localized epigenetic organization

To gain broader insights into the regulation of gene expression by PML with respect to its role in chromatin organization, the PML-regulated transcriptome was defined by RNA-seq upon PML silencing. PML knock-down led to downregulation of 1104 genes (genes positively regulated by PML) and upregulation of 1051 genes (genes negatively regulated by PML: Supplementary Figure S7A, Supplementary Table S5). Gene set enrichment analysis (Supplementary Figure S7B) revealed that genes positively regulated by PML pertain mostly to cell migration, extracellular matrix organization and EMT, in line with PML promoting metastasis in TNBC (16), and immune functions, in line with a role of PML in promoting immune responses in other cell types (7). Conversely, genes negatively regulated by PML (Supplementary Figure S7C) are predominantly implicated in metabolism, especially cholesterol biosynthetic pathways, consistent with reports of PML regulating lipid and cholesterol metabolism in other tissues (61,62). Thus, this analysis extended our previous observations (16) and showed that PML promotes expression of pro-metastatic metagenes and other gene categories that may also contribute to its oncogenic function in TNBC.

**Figure 6.**
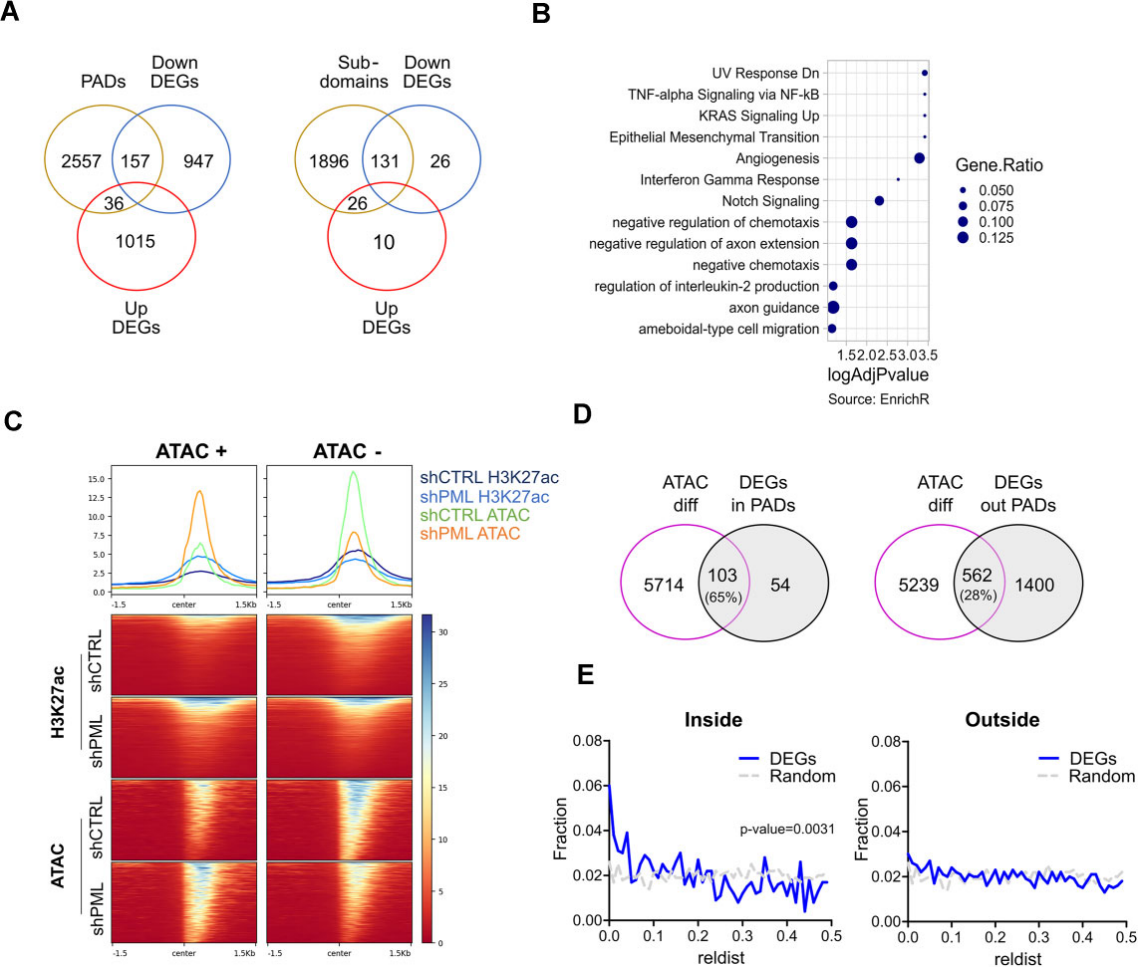
PML-modulated genes are proximal to their regulatory elements inside PADs. (**A**) Overlap of coding genes mapping to PADs (left Venn diagrams) or PADs sub-domains (right Venn diagrams) with differentially expressed genes (DEGs) upon PML silencing. DEGs are divided in downregulated (DOWN, FDR < 0.05, log_2_FC←0.5) or upregulated (UP, FDR < 0.05, log_2_FC > 0.5) genes. (**B**) Gene set enrichment analysis (EnrichR webtool) of genes downregulated upon PML silencing and mapping to PADs sub-domains. The Gene Ratio value is obtained dividing the number of observed genes for total genes contained in the indicated functional families. Results from the Fisher's exact test are represented as –log_10_(adjusted *P*-value). (**C**) ATAC-seq and H3K27ac profiles in control (shCTRL) and PML silenced (shPML) cells centered on ATAC differential peaks gaining (+) or loosing (–) accessibility upon PML silencing. (**D**) Venn diagrams of genes annotated to ATAC differential peaks and genes deregulated (DEGs) upon PML silencing inside (left) or outside (right) PADs. Percentages indicate the fraction of DEGs overlapping with genes annotated to ATAC differential peaks. (**E**) Relative distance metric between ATAC differential peaks and DEGs inside (left) or outside (right) PADs compared to the relative distance metric between randomized ATAC peaks and DEGs inside or outside PADs. Statistical significance was calculated with Kolmogorov–Smirnov test.

Amongst genes positively regulated by PML, 14% mapped to PADs, while only 3% of the negatively regulated genes fall into these regions (Figure 6A), indicating that although PADs are mostly heterochromatic, PML participates in the regulation of gene expression therein predominantly via transcriptional activation. Of note, over 80% of PAD-associated genes positively regulated by PML localize within PADs sub-domains (Figure 6A, Supplementary Table S6). Again, many of these genes are involved in migration/metastasis, immune responses and chemotaxis/axon guidance (Figure 6B). PML regulation of 3 representative pro-metastatic genes residing in PADs sub-domains (*ZEB2, PLOD2, TNC*) was confirmed in 3 TNBC cell lines (Supplementary Figure S7D) (16).

To obtain mechanistic insights into gene regulation by PML, we defined the regulatory regions of differentially expressed genes by analyzing chromatin accessibility upon PML silencing via ATAC-seq. A total of 4338 regions of differential chromatin accessibility were identified (Supplementary Figure S8A). These regions were enriched in regulatory elements (Supplementary Figure S8B) and showed coherent changes in H3K27ac peaks (Figure 6C), confirming functional regulation by PML. As expected, a fraction of differential ATAC peaks mapped to PADs (Supplementary Figure S8C). These regions were depleted of repressive marks (H3K9me3 and LMNB1) and enriched in active histone marks (H3K4me3 and H3K27ac) with respect to the rest of PADs area (Supplementary Figure S8D) and localized to PADs sub-domains (Supplementary Figure S8E). Gene annotation of ATAC peaks revealed that 65% of PML-regulated genes mapping to PADs sub-domains are within 10 kb of a differential ATAC peak, in contrast to only 28% for PML-regulated genes outside PADs (Figure 6D, Supplementary Tables S7 and S8). Moreover, distance analysis showed a closer proximity between differential ATAC peaks and PML-regulated genes within PADs than outside PADs, with randomized ATAC peaks defining baseline uniform distribution (Figure 6E). In summary, these data suggest that physical proximity between PML-regulated genes and their regulatory regions within PADs sub-domains may promote their transcriptional regulation.

Of note, beside transcriptional regulation within PADs, most genes regulated upon PML silencing localize outside PADs (Figure 6A), suggesting indirect regulation. Interestingly, TFs binding analysis performed on ATAC differential peaks (Supplementary Figure S9A) identified several oncogenes that are known to induce TNBC progression and metastasis including AP-1, c-myc and STAT3 (63–65), whose transcriptional activity is modulated by PML in other cellular systems (7,66). By analyzing a publicly available ChIP-seq dataset of STAT3 in MDA-MB-231 cells, where STAT3 is an important regulator of metastatic phenotypes (67), we found that most genes positively regulated by PML are bound by STAT3 (Figure S9B). The majority of these (690) localize outside PADs and are enriched in gene families related to metastasis (Figure S9C). Importantly, PML silencing affected STAT3 binding to the regulatory regions of 3 representative genes (*LOX*, *SPARC* and *CIITA*: Figure S9D) that are regulated by PML in TNBC cells and localize outside PADs (Figure S9E). Thus, we implicate PML in STAT3 co-activating functions in TNBC cells.

Taken together, our data indicate that in TNBC, PML regulates gene expression via a dual mechanism that involves indirect regulation of transcription and localized regulation of chromatin organization in regions of PML association.

## Discussion

Here, we show for the first time that in TNBC cells PML contributes to chromatin organization and (in)directly regulates expression of metastasis genes both within and outside domains of PML association with chromatin.

PML-associated domains (PADs) have also been described in MEFs (12). Here, we further define PADs as enriched in DNA repeats and LMNB1 and provide their functional characterization. We find that PML regulates the epigenetic composition of chromatin at a global scale by promoting maintenance of H3K9me3 deposition within PADs and restricting euchromatic marks to regions of low PML enrichment outside PADs. While this is in line with previous work that identified insulator elements at PADs borders (68), a mechanistic explanation for how PML promotes heterochromatin deposition and/or maintenance at PADs was still lacking. Association and regulation of heterochromatin by PML has been described also in other cell types and specific contexts. For example, PML associates to telomeres in mouse embryonic stem cells (69) and to viral genomes or artificial viral DNA (70,71). In these contexts, PML promotes heterochromatinization by regulating deposition of the histone variant H3.3 in cooperation with the histone chaperones DAXX and ATRX (8). In addition, in embryonic cells PML interacts and cooperates with SETDB1 (55), a histone methyltransferase that deposits H3K9me2/3 at telomeres and silences endogenous retroviral repetitive elements by promoting H3K9me3 modification and H3.3 deposition by DAXX and ATRX (72,73). Here, we confirm PML/SETDB1 interaction in TNBC cells and show that PML favors SETDB1 association to PADs, implicating PML in H3K9me3 deposition by this histone methyltransferase. Our data are consistent with SETDB1 being overexpressed and performing oncogenic and pro-metastatic functions in TNBC (54,74).

PML-mediated SETDB1 activity may be coupled to H3.3 deposition in PADs by DAXX/ATRX, which was shown in MEFs (12) and was implicated in a recent publication where SETDB1 recruitment to DAXX/H3.3/H4 complexes was suggested to be potentially mediated by PML (75). Of note, the interaction of PML with SETDB1, DAXX and ATRX was reported to occur at PML-NBs, while PADs do not colocalize with these structures, as observed here and in MEFs (12). In this respect, an important feature of PML is that it shuttles to and from the PML-NBs (6); however, it is presently unclear whether interactions with specific partner proteins established within the PML-NBs may result in relocalization of PML interactors to other nucleoplasmic locations along with PML. Thus, a functional interaction of PML with proteins involved in the heterochromatinization of PADs may either originate from the PML-NBs or occur elsewhere in the nucleoplasm. Linked to this concept is the observation that chromatin isolation techniques that were designed to enrich DNA in contact with PML-NBs, identified euchromatic and gene-rich regions (9,10,49) that are substantially different from PADs. These observations are in line with earlier studies showing that the PML-NBs are in close proximity to transcriptionally active and highly acetylated chromatin regions (76,77). Thus, we posit that PML-NBs may be especially connected to euchromatic DNA regions, while nucleoplasmic PML moieties preferentially associate to heterochromatin domains. This may appear in contrast with the reported association of PML-NBs with viral, telomeric and pericentromeric DNA regions, where PML participates to the establishment of heterochromatin (8). However, it is important to underline that these are not physiological PML-NBs, rather they represent *de novo* PML aggregates that form in response to specific pathological triggers (8). Therefore, as suggested by others, in these circumstances increased concentration of PML with affinity for heterochromatin may act as a seeding platform for the nucleation of new PML condensates with specialized functions (8).

Interestingly, we show that PML modulates the epigenome in regions that do not associate with PML. This occurs at different levels: reducing PML expression promotes not only a global increase in euchromatic marks outside PADs, but also *de novo* generation of H3K9me3 domains. We suggest that this may reflect a global reorganization of chromatin triggered by reduced deposition of H3K9me3 at PADs. This interpretation fits into the concept that chromatin homeostasis is regulated via compartmentalization and interconnection of chromatin environments (56).

Another relevant finding of our study is that embedded within the heterochromatic domains of PML association are euchromatic and gene-rich sub-domains that contain pro-metastatic genes positively regulated by PML and their regulatory regions. These regions are depleted of PML compared to remaining PADs area, thus PML does not regulate the expression of genes located therein via association to their regulatory elements. This observation may appear puzzling, but is coherent with published data demonstrating that PML regulates the expression of genes to which at large it does not associate (9,12). Also, euchromatic PADs sub-domains are enriched in genes positively rather than negatively regulated by PML. Thus, we suggest that PML regulates gene expression inside PADs by promoting the confinement of euchromatic DNA into regions of transcriptional activity. A similar function has been described for H3K9me3, which may promote gene expression inside heterochromatic domains via exclusion and confinement of actively transcribed genes (78). Specifically, mosaic distribution of H3K9me3 within heterochromatic domains allows isolation of euchromatic regions where productive interactions between regulatory elements and gene promoters drive transcription (78). Similarly, LADs contain enhancer-rich domains of low LMNB1 association and active transcription where gene expression is regulated by constraining short or long-range promoter-enhancer interactions (52,57,79,80). In sum, our results are in line with mounting evidence showing that some genes residing into constitutive heterochromatin require the surrounding repressive environment for proper expression (78).

Finally, in addition to this regulatory function on DNA, we find that PML promotes expression of genes that do not localize to PML-associated regions, suggesting indirect transcriptional regulation. This is in line with a larger body of evidence linking PML to the regulation of TFs via their expression, localization to PML-NBs and/or post-translational modifications. Examples of this literature include PML-mediated regulation of STATs (7), c-Jun (63,81) and c-Myc (82,83). Here, we provide evidence that PML may cooperate with STAT3 to promote expression of pro-metastatic genes in TNBC. This is in line with PML correlating with STAT3 signatures in metastatic breast cancer (15). As STAT3 promotes PML expression in TNBC (15), our data suggest that a PML/STAT3 positive feedback mechanism is an important regulator of TNBC progression. Overall, our data indicate that PML is a critical regulator of pro-metastatic and oncogenic metagenes in TNBC by exerting multiple and parallel transcriptional functions.

## Supplementary Material

gkad819_Supplemental_Files

## Data Availability

The dataset generated and/or analyzed during the current study are available in the Gene Expression Omnibus (GEO) at GSE226210.

## Notes

Present address: Ettore Zapparoli, Institute of Molecular Biology, Mainz, Germany.
