# Supplementary material for: PML modulates epigenetic composition of chromatin to regulate expression of pro-metastatic genes in triple-negative breast cancer
Source: Nucleic Acids Res. 2023 Oct 12;51(20):11024–39. doi: 10.1093/nar/gkad819 (PMC10639071; doi:10.1093/nar/gkad819)
Supplement: gkad819_Supplemental_Files [file gkad819_supplemental_files.zip › Fracassi et al Supplementary.pdf]

Supplementary Data

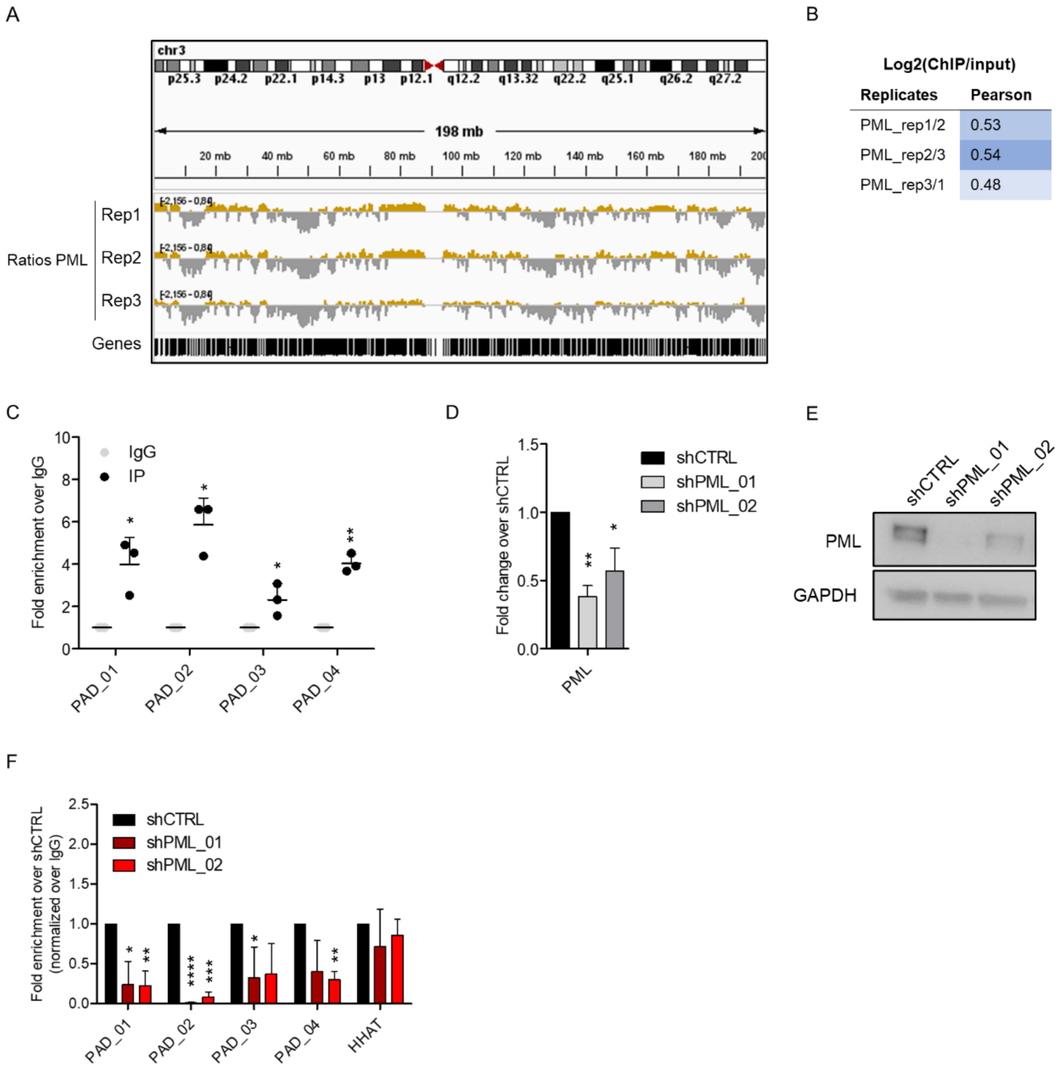

Supplementary Figure S1. PML-associated domains in MDA-MB-231 cells. A. Genome browser view of PML ChIP enrichment values expressed as Log2(ChIP/input) ratios across chromosome 3 in each biological replicate (Rep; *y* axis range shown in brackets). **B.** Pearson correlations of Log2(PML ChIP/input) ratios across the genome between replicates. **C.** Association of PML to 4 randomly selected intra-PAD sequences via ChIP-qPCR. Shown are mean values ± SD of 3 biological replicates represented as fold enrichment over control IgG. Statistical significance was determined by paired Student's t-test. *P<5*10^{-2}, **P<10^{-2}, ***P<10^{-3}. **D.** RT-qPCR analysis of PML transcripts in cells silenced with a control shRNA (shCTRL) and two shRNAs against PML (shPML_01 and shPML_02). Relative expression levels of PML were expressed as fold change values of control cells (shCTRL). Data represent mean values ± SD of three independent experiments. Statistical significance was calculated with paired Student's *t*-test; *P<5*10^{-2}, **P<10^{-2}. **E.** Western blot analysis of PML in cells silenced with a control shRNA (shCTRL) and two shRNAs against PML (shPML_01 and shPML_02). GAPDH was used as loading control. **F.** Association of PML to 4 intra-PAD sequences and the *HHAT* gene localizing outside PADs via ChIP-qPCR in cells silenced with a control shRNA (shCTRL) and two shRNAs against PML (shPML_01 and shPML_02). Shown are mean values ± SD of 3 biological replicates normalized over control IgG and represented as fold enrichment over shCTRL. Statistical significance was determined by paired Student's t-test. *P<5*10^{-2}, **P<10^{-2}, ***P<10^{-3}, ***P<10^{-4}.

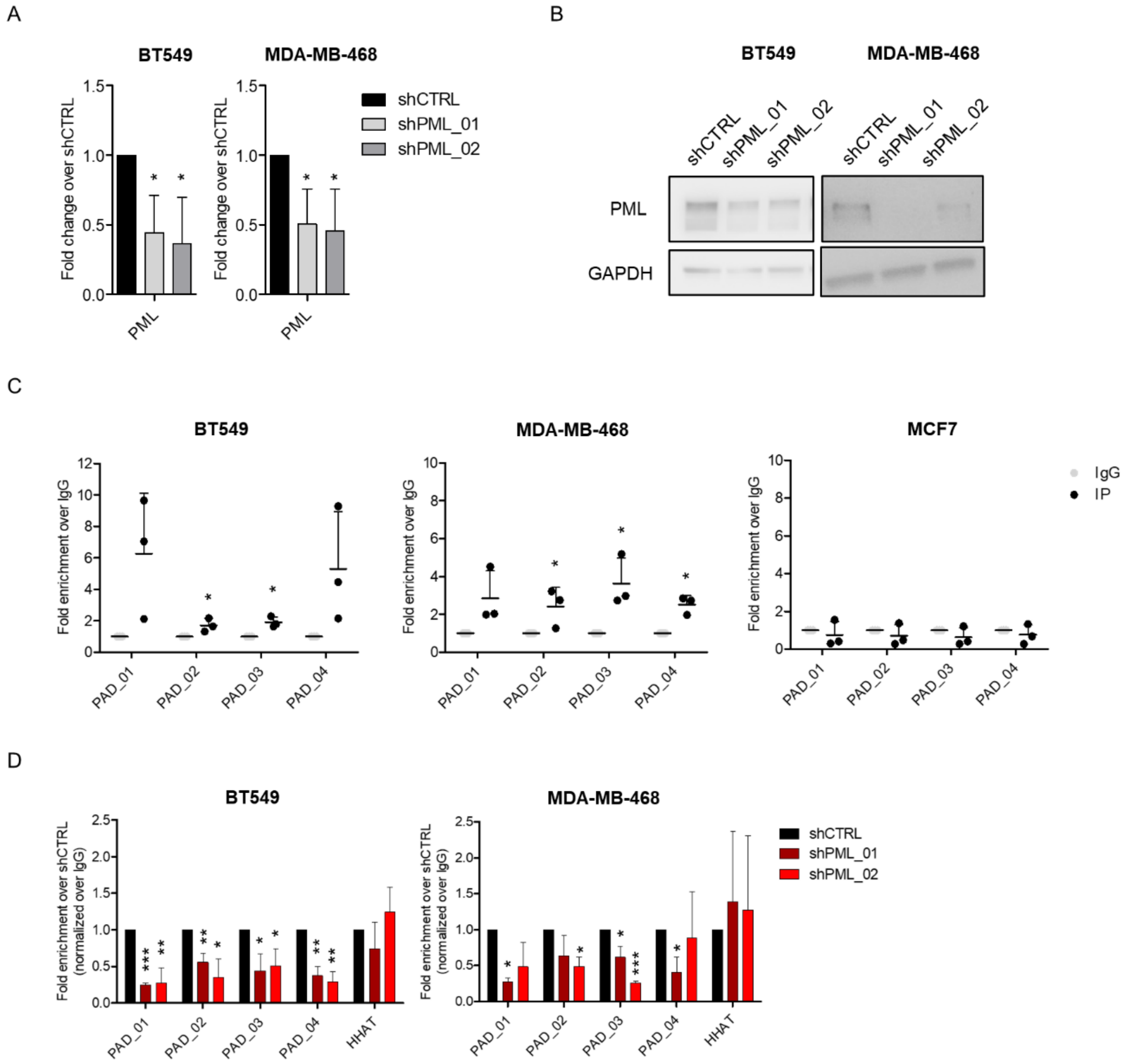

Supplementary Figure S2. Validation of PML association to PADs in TNBC cell lines. A. RT-qPCR analysis of PML transcripts in BT549 and MDA-MB-468 cells silenced with a control shRNA (shCTRL) and two shRNAs against PML (shPML_01 and shPML_02). Relative expression levels of PML were expressed as fold change values of control cells (shCTRL). Data represent mean values ± SD of three independent experiments. Statistical significance was determined by paired Student's t-test. $*P<5*10^{-2}$. **B.** Western blot analysis of PML in BT549 and MDA-MB-468 cells silenced with a control shRNA (shCTRL) and two shRNAs against PML (shPML_01 and shPML_02). GAPDH was used as loading control. **C.** Association of PML to 4 randomly selected intra-PAD sequences via ChIP-qPCR in BT549, MDA-MB-468 and MCF7 cells. Shown are mean values ± SD of 3 biological replicates represented as fold enrichment over control IgG. Statistical significance was determined by paired Student's t-test. $*P<5*10^{-2}$. **D.** Association of PML to 4 intra-PAD sequences and the *HHAT* gene localizing outside PADs via ChIP-qPCR in BT549 and MDA-MB-468 cells silenced with a control shRNA (shCTRL) and two shRNAs against PML (shPML_01 and shPML_02). Shown are mean values ± SD of 3 biological replicates normalized over control IgG and represented as fold enrichment over shCTRL. Statistical significance was determined by paired Student's t-test. $*P<5*10^{-2}$, $**P<10^{-2}$, $***P<10^{-3}$.

A

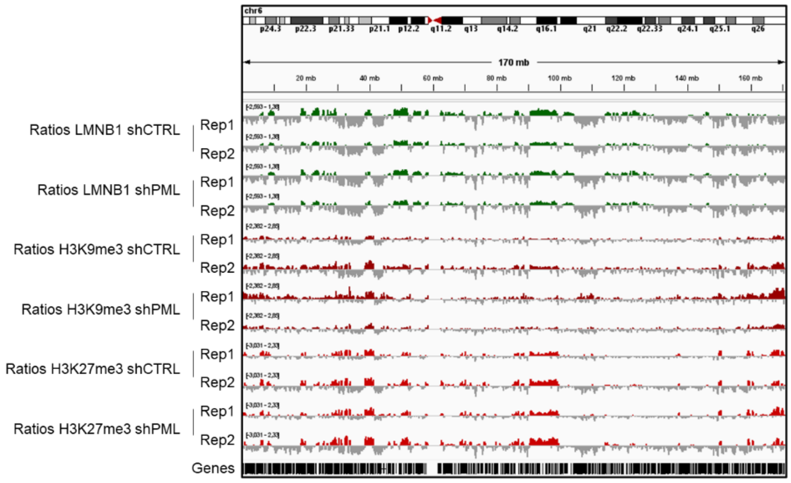

B

Log2(ChIP/input)

ChIP	Condition	Pearson
LMNB1	shCTRL	0.84
	shPML	0.86
H3K9me3	shCTRL	0.71
	shPML	0.71
H3K27me3	shCTRL	0.70
	shPML	0.75

C

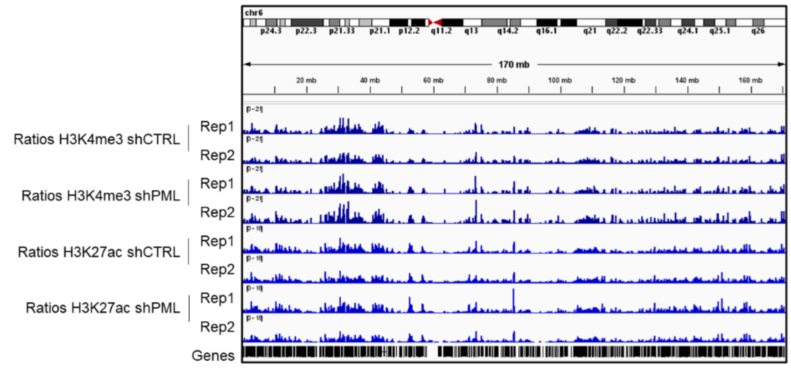

D

Normalized counts

ChIP	Condition	Pearson
H3K4me3	shCTRL	0.95
	shPML	0.95
H3K27ac	shCTRL	0.92
	shPML	0.96

E

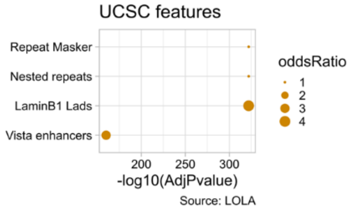

F

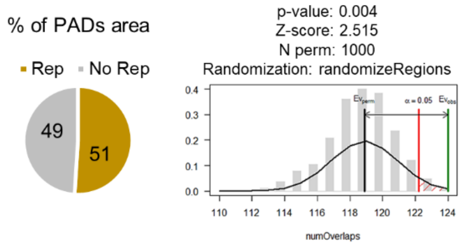

G

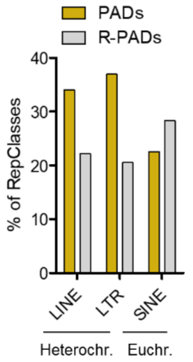

Supplementary Figure S3. Profiles of ChIP-seq enrichments among replicates and genomic features of PADs. A, C. Genome browser views of the indicated ChIP enrichment values expressed as Log2(ChIP/input) ratios and normalized counts across chromosome 6 in each biological replicate (Rep) in control (shCTRL) and PML silenced (shPML) cells (*y* axis range shown in brackets). **B, D.** Pearson correlations of Log2(ChIP/input) ratios and normalized count values across the genome between replicates in shCTRL and shPML cells. **E.** Enrichment Analysis (via LOLAweb interactive server) of genomic features annotated by the UCSC database in PADs. Results from the Fisher's exact test are represented as -log10(Adjusted p-value) and odds ratio. **F.** Percentage of PADs area covered by repetitive (Rep) and not repetitive (No Rep) elements. Permutation tests on the right reveal statistical significance of overlap. **G.** Percentage of repetitive elements (RepClasses) enriched in heterochromatic (Heterochr.) and euchromatic (Euchr.) DNA regions mapping to PADs and random PADs (R-PADs).

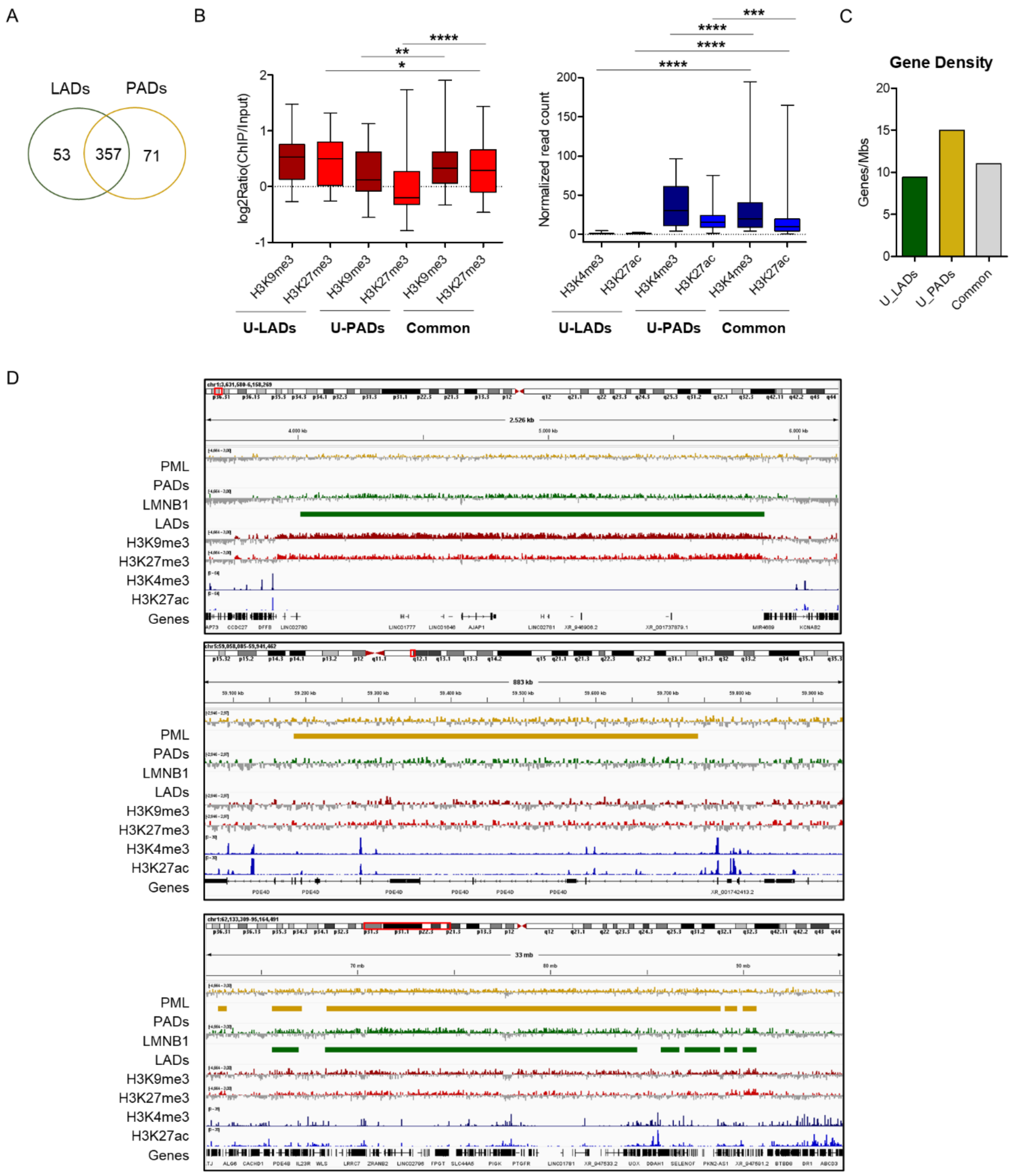

Supplementary Figure S4. Analysis of PADs and LADs differential features. A. Venn diagrams of LADs and PADs. **B.** Enrichment in unique LADs (U-LADs), unique PADs (U-PADs) and domains of PML/LMNB1 association (Common) of H3K9me3 and H3K27me3 (left graph), H3K4me3 and H3K27ac (right graph); bar, median; whiskers, min-max; *P<5*10^{-2}, **P<10^{-2}, ***P<10^{-3}, ****P<10^{-4}; unpaired t-tests with Welch's correction. **C.** Gene density in U-LADs, U-PADs and Common domains expressed and genes/mb. **D.** Genome browser views of the indicated ChIP enrichment values (*y* axis range shown in brackets) across U-LADs (upper panel), U-PADs (middle panel) and Common domains of PML and LMNB1 association (lower panel). All data were obtained in MDA-MB-231 cells.

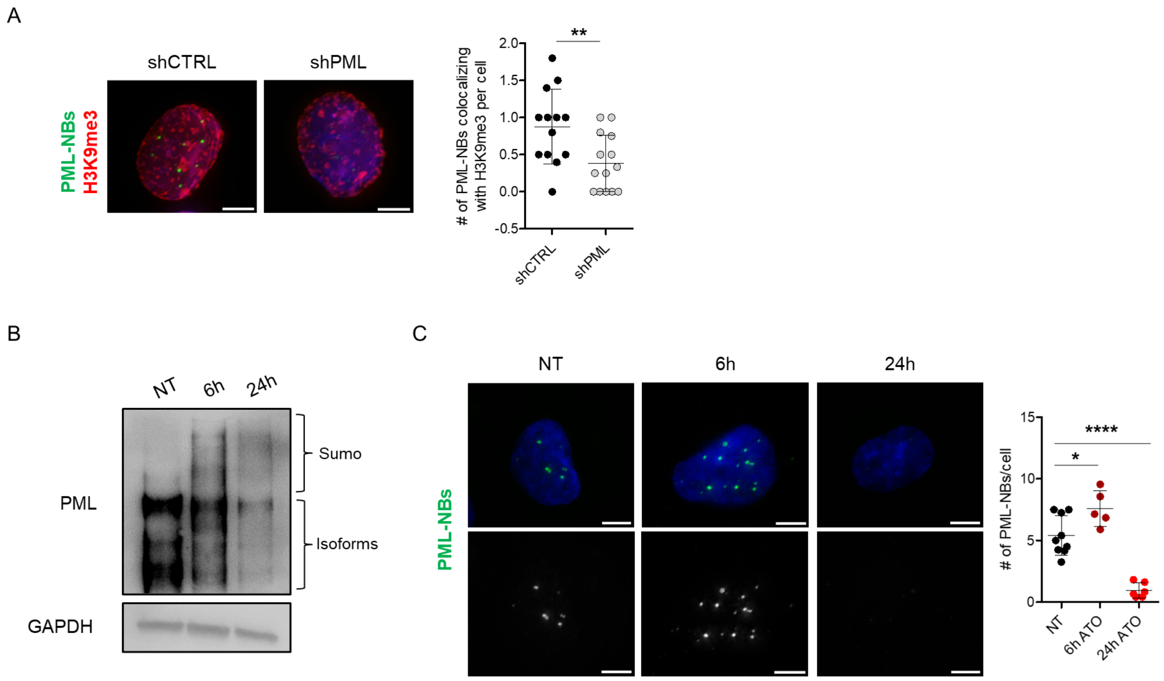

Supplementary Figure S5. Analysis of PML proximity to H3K9me3 foci and effect of arsenic trioxide on PML. A. Immunofluorescence of PML (green signal) and H3K9me3 (red signal) in control (shCTRL) and PML silenced (shPML) cells. Graph on the right indicates the number of PML-NBs colocalizing with H3K9me3 foci/cell in control (shCTRL) and PML silenced (shPML) cells. A total of 50 nuclei/conditions were analyzed. DNA was stained with DAPI (blue). Images are compressed z-stacks. Bar, 5 μm. Data represent mean values ± SD. Statistical significance was determined by unpaired Student's t-test. ***P*$<10^{-2}$. **B.** Western blot analyses of PML in untreated cells (NT) and upon treatment with 1 μM arsenic trioxide (ATO) for 6 or 24 hours. GAPDH was used as loading control. **C.** Immunofluorescence of PML (green signal) in NT cells and upon treatment with 1 μM ATO for 6 or 24 hours. Graph on the right indicates the number of PML-NBs/cell. A total of 40 nuclei/conditions were analyzed. DNA was stained with DAPI (blue). Images are compressed z-stacks. Bar, 5 μm. Data represent mean values ± SD. Statistical significance was determined by unpaired Student's t-test. **P*$<5*10^{-2}$, *****P*$<10^{-4}$. All data were obtained in MDA-MB-231 cells.

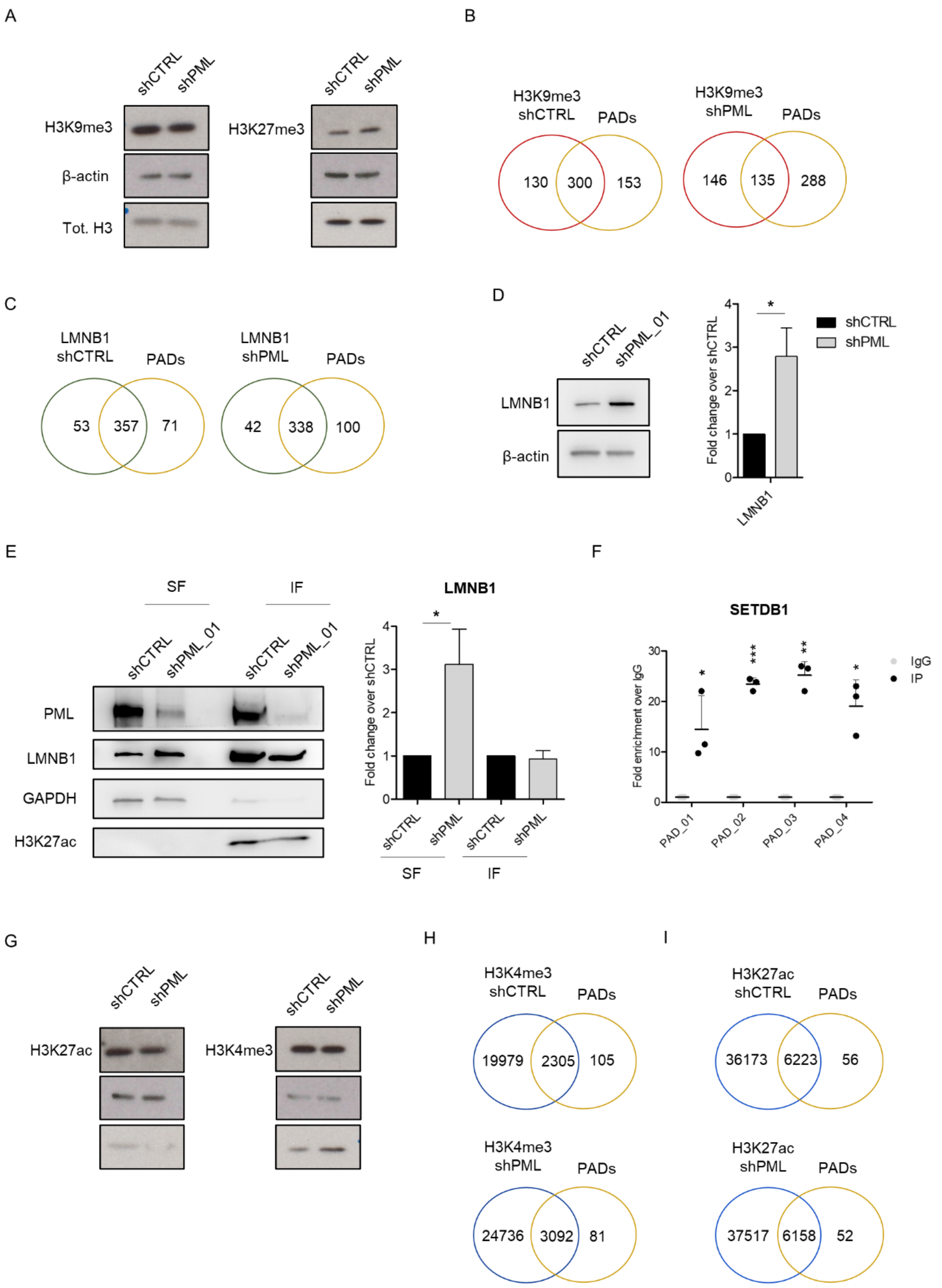

Supplementary Figure S6. Regulation of histone post-translational modifications and LMNB1 by PML. A. Western blot analyses of H3K9me3 and H3K27me3 in control (shCTRL) and cells silenced for PML (shPML). β-actin and total histone H3 were used as controls. **B, C.** Venn diagrams of overlapping H3K9me3 (**B**) or LMNB1 (**C**) domains with PADs in control (shCTRL) and cells silenced for PML (shPML). **D.** Western blot analysis of LMNB1 in control (shCTRL) and cells silenced for PML (shPML). β-actin was used as control. Right graph, densitometric analysis of LMNB1 western blot in shCTRL and shPML cells. Data are normalized on β-actin and expressed as fold change over control cells (shCTRL). Shown are mean values ± SD of 3 biological replicates

represented as fold enrichment over shCTRL. Statistical significance was determined by paired Student's t-test. *$P<5*10^{-2}$. **E.** Western blot analysis of LMNB1 and PML upon nuclear fractionation into soluble fraction (SF) and insoluble fraction (IF) in control (shCTRL) and cells silenced for PML (shPML). GAPDH and H3K27ac were used as loading controls. Right graph, densitometric analysis of LMNB1 in the nuclear fractions in shCTRL and shPML cells. Shown are mean values ± SD of 3 biological replicates represented as fold enrichment over shCTRL. Statistical significance was determined by paired Student's t-test. *$P<5*10^{-2}$. **F.** Association of SETDB1 to 4 randomly selected intra-PAD sequences via ChIP-qPCR. Shown are mean values ± SD of 3 biological replicates represented as fold enrichment over control IgG. Statistical significance was determined by paired Student's t-test. *$P<5*10^{-2}$, **$P<10^{-2}$, ***$P<10^{-3}$. **G.** Western blot analyses of H3K27ac and H3K4me3 in control (shCTRL) and cells silenced for PML (shPML). β-actin and total histone H3 were used as controls. Representative western blots of (**G**) and (**A**) derive from the same protein extracts loaded on different lanes. **H, I.** Venn diagrams of overlapping H3K27ac (**H**) and H3K4me3 (**I**) peaks with PADs in control (shCTRL) and cells silenced for PML (shPML). All data were obtained in MDA-MB-231 cells.

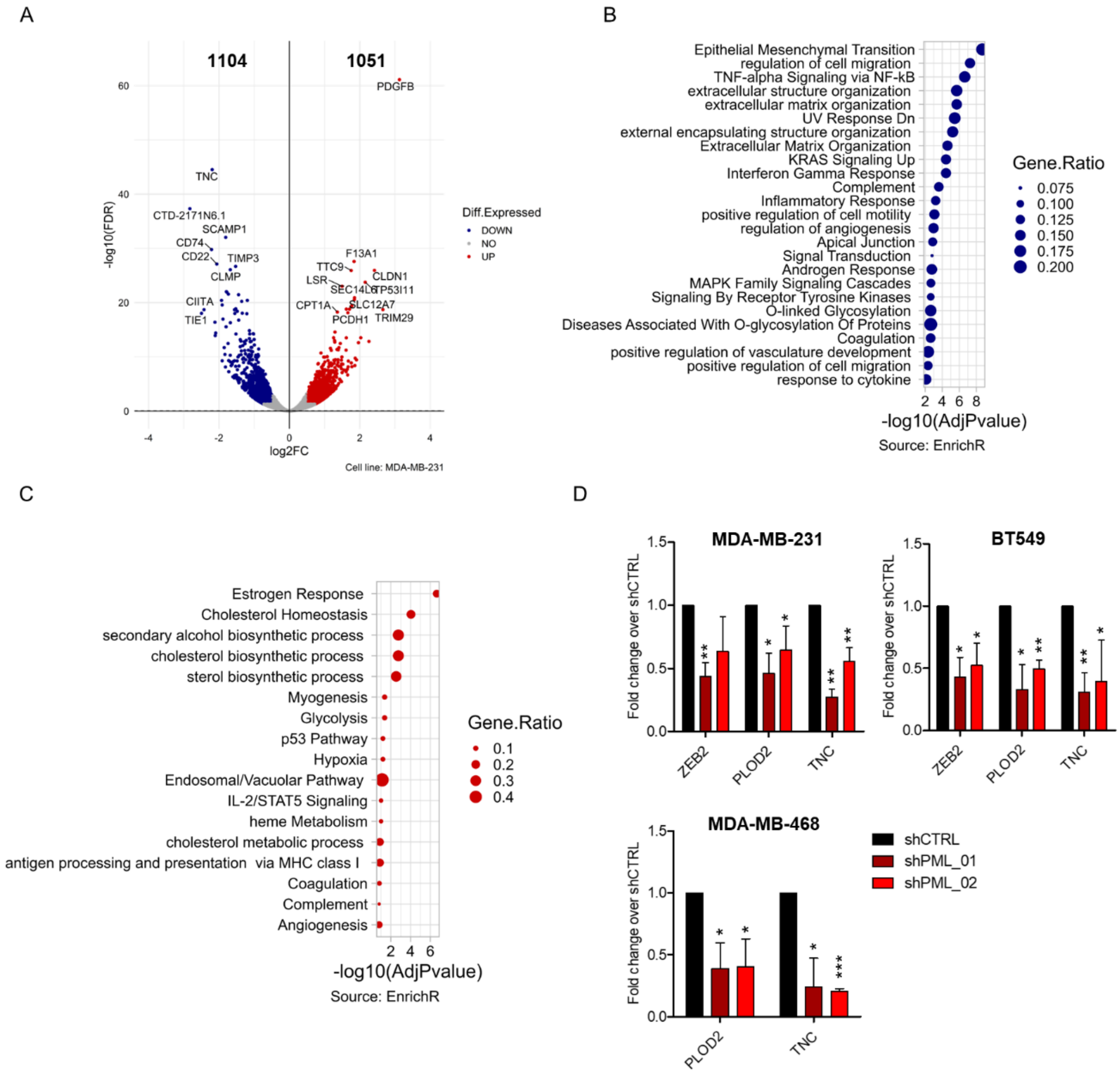

Supplementary Figure S7. The PML regulated transcriptome in TNBC cells. A. Volcano plot of genes deregulated in response to PML silencing and identified via RNA-seq. The x and y axis represent log2Fold change (FC) and -log10 False discovery rate (FDR) values, respectively. Blue dots represent downregulated (DOWN) genes with log2FC<-0.5 and red dots upregulated (UP) genes with log2FC>0.5. **B, C.** Gene set enrichment analysis (EnrichR webtool) of genes downregulated (**B**) and upregulated (**C**) upon PML silencing. The Gene Ratio value is obtained dividing the number of observed genes for total genes contained in the indicated functional families. Results from the Fisher's exact test are represented as -log10(Adjusted p-value). Data in **A-C** were obtained in MDA-MB-231 cells. **D.** RT-qPCR analysis of genes positively regulated by PML and involved in metastasis in MDA-MB-231, BT549 and MDA-MB-468 cells. ZEB2 expression was not observed in MDA-MB-468 cells. Relative expression levels of each gene were compared to shCTRL cells. Data represent mean values ± SD of three independent experiments. Statistical significance was determined by paired Student's t-test. **P*<5*10^{-2}, ***P*<10^{-2}, ****P*<10^{-3}.

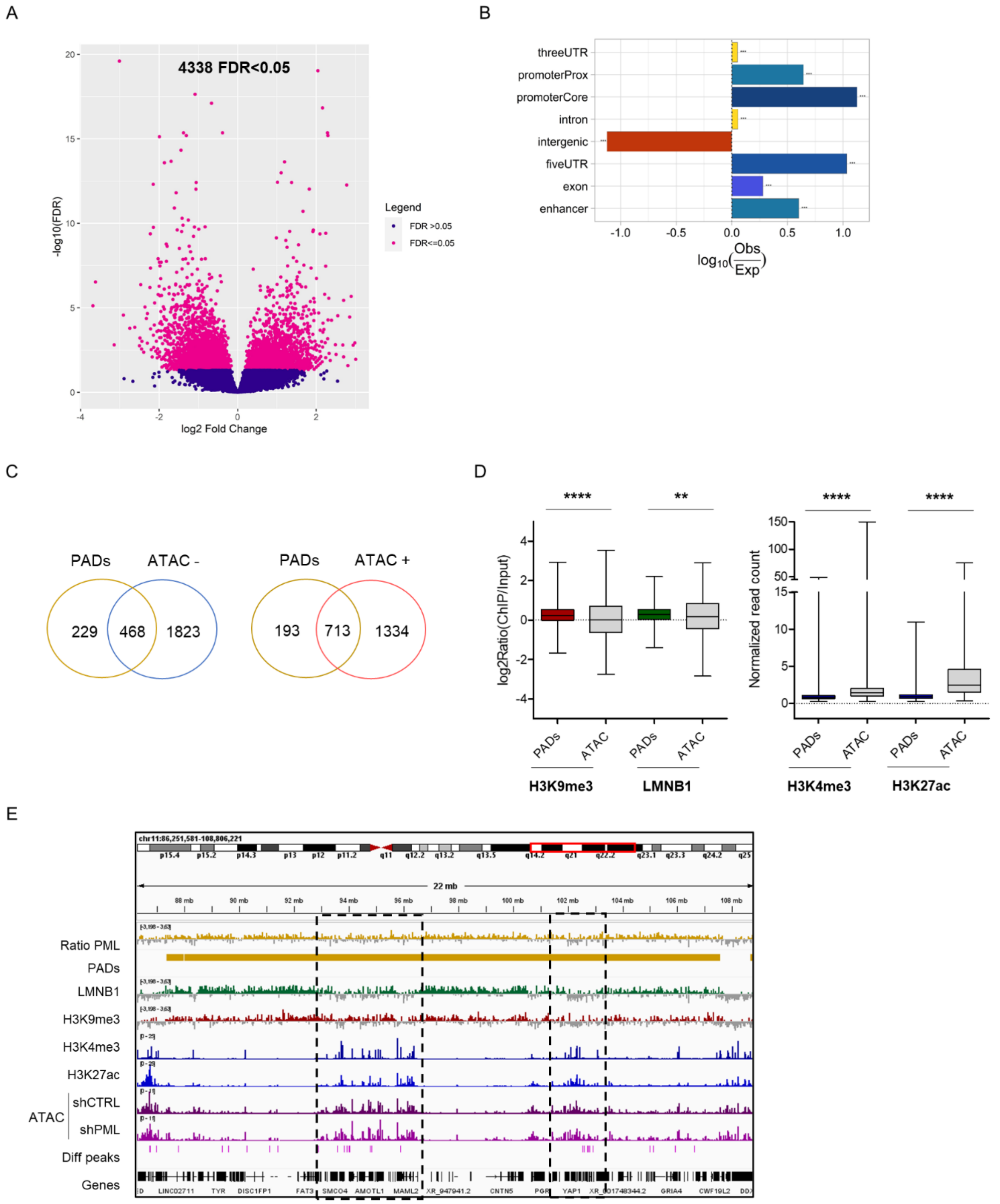

Supplementary Figure S8. Regulation of chromatin accessibility by PML in MDA-MB-231 cells. **A.** Volcano plot of differential ATAC-seq peaks in response to PML silencing. The x and y axis represent log2Fold change (FC) and -log10 False discovery rate (FDR) values, respectively. Pink dots represent significant peaks with FDR</>0.5. **B.** Expected partition distribution of ATAC differential peaks. **C.** Venn diagrams of overlapping PADs and decreased (-) or increased (+) ATAC accessibility in cells silenced for PML (shPML) versus control (shCTRL) cells. **D.** Enrichment of H3K9me3 and LMNB1 (left), H3K4me3 and H3K27az (right) in PADs and ATAC peaks; bar, median; whiskers, min-max; $^{**}P<10^{-2.}$, $^{****}P<10^{-4}$; unpaired t-tests with Welch's correction. **E.** Genome browser view of Log2(ChIP/input) ratios and normalized counts (*y* axis range shown in brackets) of PML, called PADs, LMNB1, H3K9me3, H3K4me3, H3K27ac, ATAC in shCTRL and shPML cells and differential ATAC peaks.

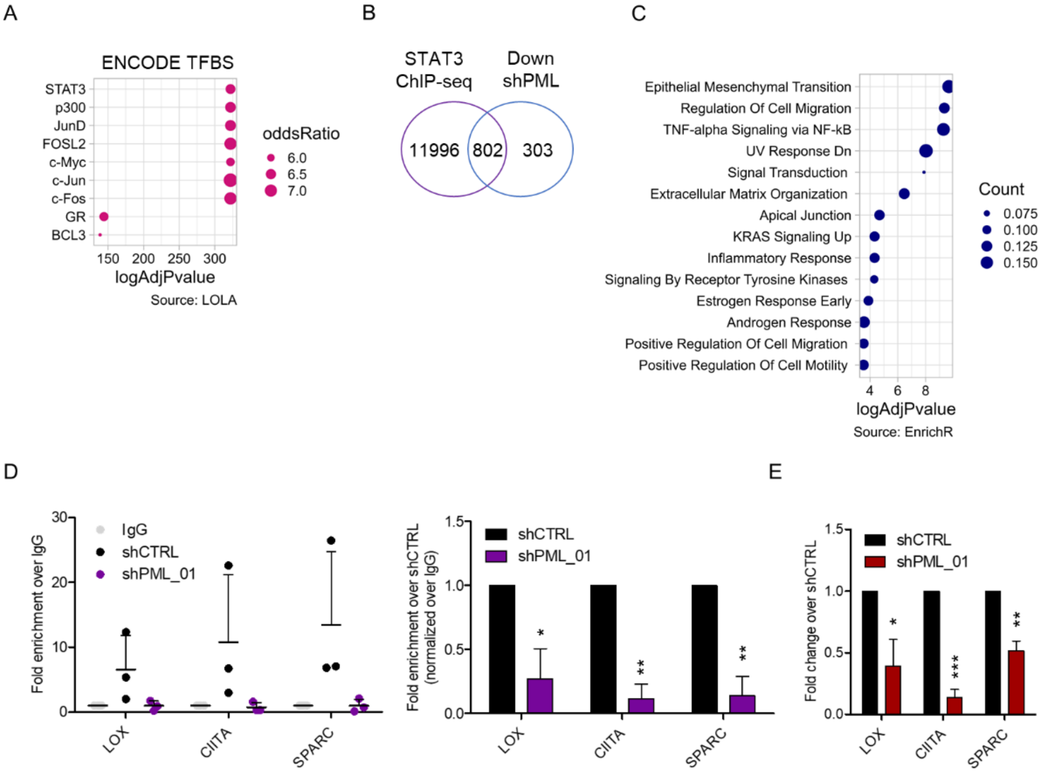

Supplementary Figure S9. PML regulates STAT3 association to target genes in MDA-MB-231 cells. A. Enrichment analysis (via LOLAweb interactive server) of transcription factors binding sites mapped by Encode on ATAC differential peaks obtained upon PML silencing. Results from the Fisher's exact test are represented as -log10(Adjusted p-value) and odds ratio. **B.** Venn diagram of annotated genes for STAT3 ChIP-seq and genes positively regulated by PML (downregulated upon PML silencing). **C.** Gene set enrichment analysis (EnrichR webtool) of genes bound by STAT3 and positively regulated by PML. The Gene Ratio value is obtained dividing the number of observed genes for total genes contained in the indicated functional families. Results from the Fisher's exact test are represented as -log10(Adjusted p-value). **D.** Validation of STAT3 association to 3 genes positively regulated by PML and localizing outside PADs via ChIP-qPCR. Shown are mean values ± SD of 3 biological replicates represented as fold enrichment over control IgG (upper panel), and fold enrichment of PML-silenced cells over shCTRL (lower panel). Statistical significance was determined by paired Student's t-test. $*P<5*10^{-2}$, $**P<10^{-2}$. **E.** RT-qPCR analysis of genes positively regulated by PML and bound by STAT3 in control (shCTRL) and PML-silenced (shPML) MDA-MB-231 cells. Relative expression levels of each gene were compared to shCTRL cells. Data represent mean values ± SD of three independent experiments. Statistical significance was calculated with paired Student's *t*-test. $*P<5*10^{-2.}$, $**P<10^{-2.}$, $***P<10^{-3}$.

Supplementary Tables (uploaded separately)

Table S1. Description of domains and peaks identified in control and PML silenced TNBC cells.
Table S2. Observed overlaps and coverages of HPTMs and LADs with PADs.
Table S3. Genes falling in PADs.
Table S4. Gene annotation of PADs subdomains.
Table S5. Genes deregulated upon PML silencing.
Table S6. Genes deregulated upon PML silencing and falling in PADs subdomains.
Table S7. Gene annotation of differential ATAC-seq peaks.
Table S8. Genes deregulated upon PML silencing, inside and outside PADs, and proximal to an ATAC differential peak.

Supplementary File 2

```
clear; close all;


FISHTable = readtable('Ch3_FISH-QUANT__all_spots_230606.txt',...
   'HeaderLines', 13, 'ReadVariableNames', 1);


PMLTable = readtable('Ch2_FISH-QUANT__all_spots_230606.txt',...
   'HeaderLines', 13, 'ReadVariableNames', 1);


for iFISH= 1: height(FISHTable)

   if FISHTable.in_Nuc(iFISH)

      X0 = FISHTable.Pos_X(iFISH);
      Y0 = FISHTable.Pos_Y(iFISH);
      Z0 = FISHTable.Pos_Z(iFISH);

      FileName = FISHTable.File(iFISH);
      FileName = strrep(FileName, 'CH3_', 'CH2_');

      Cell =  FISHTable.Cell(iFISH);

      idx = find(ismember(PMLTable.File,FileName) & ismember(PMLTable.Cell,Cell)&
PMLTable.in_Nuc);
      if isempty(idx)
        FISHTable.minDistanceToPML(iFISH) = NaN;
      else

         Distances = ((PMLTable.Pos_X(idx) - X0).^2 + ...
            (PMLTable.Pos_Y(idx) - Y0).^2 + ...
            (PMLTable.Pos_Z(idx) - Z0).^2).^0.5;
         FISHTable.minDistanceToPML(iFISH) = min(Distances);
      end



   else
      FISHTable.minDistanceToPML(iFISH) = NaN;
   end

end

%%
writetable(FISHTable, 'FISHTable_WithDistanceFromPML.xls');
```
